# Yellow fever virus is susceptible to sofosbuvir both *in vitro* and *in vivo*

**DOI:** 10.1371/journal.pntd.0007072

**Published:** 2019-01-30

**Authors:** Caroline S. de Freitas, Luiza M. Higa, Carolina Q. Sacramento, André C. Ferreira, Patrícia A. Reis, Rodrigo Delvecchio, Fabio L. Monteiro, Giselle Barbosa-Lima, Harrison James Westgarth, Yasmine Rangel Vieira, Mayara Mattos, Natasha Rocha, Lucas Villas Bôas Hoelz, Rennan Papaleo Paes Leme, Mônica M. Bastos, Gisele Olinto L. Rodrigues, Carla Elizabeth M. Lopes, Celso Martins Queiroz-Junior, Cristiano X. Lima, Vivian V. Costa, Mauro M. Teixeira, Fernando A. Bozza, Patrícia T. Bozza, Nubia Boechat, Amilcar Tanuri, Thiago Moreno L. Souza

**Affiliations:** 1 Laboratório de Imunofarmacologia, Instituto Oswaldo Cruz (IOC), Fundação Oswaldo Cruz (Fiocruz), Rio de Janeiro, RJ, Brazil; 2 National Institute for Science and Technology on Innovation on Neglected Diseases (INCT/IDN), Center for Technological Development in Health (CDTS), Fiocruz, Rio de Janeiro, RJ, Brazil; 3 Laboratório de Virologia Molecular, Instituto de Biologia, Universidade Federal do Rio de Janeiro (UFRJ), Rio de Janeiro, RJ, Brazil; 4 Instituto Nacional de Infectologia (INI), Fiocruz, Rio de Janeiro, RJ, Brazil; 5 Instituto de Tecnologia de Fármacos (Farmanguinhos), Fiocruz, Rio de Janeiro, RJ, Brazil; 6 Center for Research and Development of Pharmaceuticals, Institute of Biological Sciences (ICB), Universidade Federal de Minas Gerais (UFMG), Minas Gerais, Brazil; 7 Research Group in Arboviral Diseases, Department of Morphology, Institute of Biological Sciences (ICB), Universidade Federal de Minas Gerais (UFMG), Minas Gerais, Brazil; 8 Cardiac Lab, Department of Morphology, Institute of Biological Sciences (ICB), Universidade Federal de Minas Gerais (UFMG), Minas Gerais, Brazil; 9 Departamento de Cirurgia, Faculdade de Medicina, Universidade Federal de Minas Gerais, Belo Horizonte, Minas Gerais, Brazil; 10 Immunopharmacology Lab, Department of Biochemistry and Immunology, Institute of Biological Sciences (ICB), Universidade Federal de Minas Gerais (UFMG), Minas Gerais, Brazil; Northeastern University, UNITED STATES

## Abstract

Yellow fever virus (YFV) is a member of the *Flaviviridae* family. In Brazil, yellow fever (YF) cases have increased dramatically in sylvatic areas neighboring urban zones in the last few years. Because of the high lethality rates associated with infection and absence of any antiviral treatments, it is essential to identify therapeutic options to respond to YFV outbreaks. Repurposing of clinically approved drugs represents the fastest alternative to discover antivirals for public health emergencies. Other Flaviviruses, such as Zika (ZIKV) and dengue (DENV) viruses, are susceptible to sofosbuvir, a clinically approved drug against hepatitis C virus (HCV). Our data showed that sofosbuvir docks onto YFV RNA polymerase using conserved amino acid residues for nucleotide binding. This drug inhibited the replication of both vaccine and wild-type strains of YFV on human hepatoma cells, with EC_50_ values around 5 μM. Sofosbuvir protected YFV-infected neonatal Swiss mice and adult type I interferon receptor knockout mice (A129^-/-^) from mortality and weight loss. Because of its safety profile in humans and significant antiviral effects *in vitro* and in mice, Sofosbuvir may represent a novel therapeutic option for the treatment of YF. **Key-words:** Yellow fever virus; Yellow fever, antiviral; sofosbuvir

## Author summary

Yellow fever virus is transmitted by mosquitoes and its infection may be asymptomatic or lead to a wide clinical spectrum ranging from a mild febrile illness to a potentially lethal viral hemorrhagic fever characterized by liver damage. Although a yellow fever vaccine is available, low coverage allows 80,000–200,000 cases and 30,000–60,000 deaths annually worldwide. There are no specific therapy and treatment relies on supportive care, reinforcing an urgent need for antiviral repourposing. Here, we showed that sofosbuvir, clinically approved against hepatitis C, inhibits yellow fever virus replication in liver cell lines and animal models. In vitro, sofosbuvir inhibits viral RNA replication, decreases the number of infected cells and the production of infectious virus particles. These data is particularly relevante since the liver is the main target of yellow fever infection. Sofosbuvir also protected infected animals from mortality, weight loss and liver injury, especially prophylatically. Our pre-clinical results supports a second use of sofosbuvir against yellow fever.

## Introduction

Yellow fever virus (YFV) is a single-strand positive-sense RNA virus which belongs to the *Flaviviridae* family. Yellow fever (YF) outbreaks were very common throughout the tropical world until the beginning of the 20^th^ century, when vaccination and vector control limited the urban virus circulation [1]. Classically, sylvatic and urban cycles of YFV transmission occur. Non-human primates are sylvatic reservoirs of jungle YFV and non-immunized humans entering the rain forest and those living in the ecotone (between preserved rain forest and urban area) are highly susceptible to YFV, which is transmitted by mosquitoes from *Haemagogus* and *Sabethes* genera [2]. The virus is usually brought to urban settings by viremic humans infected in the jungle [2]. The urban cycle involves transmission of the virus among humans by vectors like *Aedes* spp. mosquitoes [2].

Brazil, an endemic country for YF, failed to vaccinate a large proportion of the susceptible population. This scenario of low human vaccinal coverage along with increased sylvatic YFV activity in primates has been occurring in Brazil since 2016, leading to bursts of human cases of YF. For instance, between the second semester of 2017 and March 2018, 4,847 epizootics were reported and 920 human cases were confirmed. There were 300 deaths associated with this outbreak [3, 4]. In fact, cases of YF increased 1.8-times compared to the previous 35 years [3]. Altogether, these data also show that YFV spread from Brazilian rain forests to the outskirt of major cities in the Southeastern region of the country. Despite the detection of YFV in some urban areas in humans and primates during this recent reemergence, the Brazilian Ministry of Health (MoH) has argued this was a sylvatic cycle with no urban autochthonous transmission. Indeed, most of the recent activity of YFV was observed in areas adjacent to the Atlantic forest, where the genotype I was introduced two times in 2005 (95% interval: 2002–2007) and 2016 (95% interval: 2012–2017), spilling over from the Amazon forest [5].

YFV causes massive lethality in new world monkeys and around 30% in humans [6]. Once displaying signs of severe infection, such as bleeding, shock, liver function decay and jaundice, infected individuals are likely to progress to poor clinical outcomes. Most often, acute hepatic failure occurs rapidly. No specific treatment options to YFV exist and patients solely receive intensive palliative care. Therefore, antivirals with anti-flavivirus activity may represent an important alternative for drug repurposing in an attempt to improve patient outcome.

Developed in the 1930s, YF 17D live-attenuated vaccine confers long-lasting immunity to its recipients. Vaccination is recommended for individuals aged ≥ 9 months who are living in or travelling to areas at risk for YF. Contraindications include hypersensitivity to vaccine components, severe immunodeficiency, and age under ≤6 months [7]. Even though 17D is highly effective and one of the safest vaccines in history, rare severe adverse events have been reported. YF vaccine-associated neurological (YEL-AND) and viscerotropic (YEL-AVD) diseases are similar to classic YF caused by wild-type (WT) virus. Reporting rates of YEL-AND and YEL-AVD are 0.8 and 0.4 cases per 100,000 doses distributed [8]. Specific treatment would be of utmost importance for individuals with YF vaccine-associated diseases and for YFV-exposed people for whom vaccination is contraindicated.

Our group and others have recently shown that sofosbuvir, a clinically approved anti-hepatitis C virus (HCV) drug, is also endowed with anti-Zika virus (ZIKV) antiviral activity *in vitro* and *in vivo* [9–11]. It has also been shown that sofosbuvir also blocks dengue virus (DENV) replication [12]. Animal model studies of sofosbuvir on Flaviviruses reveal that this drug is more effective when used prophylactically or as early as possible. Sofosbuvir was approved by the Food and Drug Administration (FDA) in 2013. It has been used in therapy regimens thereafter in large worldwide scale to treat HCV-infected individuals, with infrequent registers of toxicity and adverse effects even for complex patients, such as those co-infected with both HIV/HCV or with substantial liver damage provoked by HCV [13–15]. Sofosbuvir is very effective against HCV genotype 1/2/3, and safe doses may range from 400 to 1200 mg daily for up to 24 weeks [13–15]. When compared to pan-antiviral drugs such as ribavirin, sofosbuvir is considered safer for pregnant woman [16, 17].

In the intention to have a safe and an active antiviral compound to inhibit YFV replication, we tested whether the virus was susceptible to sofosbuvir. We found that sofosbuvir binds to conserved amino acid residues on the YFV RNA polymerase (NS5), inhibiting virus replication in human hepatoma cells, diminishing YFV-associated mortality and improving the hepatic condition in animal models.

## Materials and methods

### Reagents

The antiviral sofosbuvir was kindly donated by Dr. Jaime Rabi (Microbiológica Química e Farmacêutica, Brazil; part of the BMK Consortium). Ribavirin and AZT were provided by Instituto de Tecnologia de Farmacos (Farmanguinhos, Fiocruz). Drugs were dissolved in 100% dimethylsulfoxide (DMSO) and subsequently diluted at least 10^4^-fold in culture medium before each assay. The final DMSO concentration showed no cytotoxicity. The materials for cell culture were purchased from Thermo Scientific Life Sciences (Grand Island, NY) unless otherwise mentioned.

### Cells and virus

Human hepatoma cell lines (Huh-7 and HepG2) and African green monkey (Vero) cells were cultured in DMEM supplemented with 10% fetal bovine serum (FBS; HyClone, Logan, Utah), 100 U/mL penicillin, and 100 μg/mL streptomycin [18, 19]. Cells were incubated at 37°C in 5% CO_2_. *Aedes albopictus* C6/36 cells were cultured at 28°C in Leibovitz L15 medium supplemented with 2 mM L-glutamine, 0.75 g/L sodium bicarbonate, 0.3% tryptose phosphate broth, non-essential amino acids and 5% FBS.YFV vaccinal and WT strains were passaged at an multiplicity of infection (MOI) of 0.01 in either Vero (for 24–72 h at 37°C) or C6/36 (for 6 days at 28°C) cells. Virus titers were also determined in Vero cell cultures by TCID_50_/mL [20] or plaque forming units (PFU)/mL (described below). The vaccine strain 17D was donated by the Reference Laboratory for Flavivirus, Fiocruz, Brazilian Ministry of Health, whereas the WT strain was isolated in Vero cells from the serum of a symptomatic patient with confirmed RT-PCR result for YFV (GenBank accession #MH018072) [21].

### Cytotoxicity assay

Monolayers of 1.5 x 10^4^ hepatoma cells in 96-well plates were treated for 5 days with various concentrations of sofosbuvir or ribavirin as a control. Then, 5 mg/ml 2,3-bis-(2-methoxy-4-nitro-5-sulfophenyl)-2*H*-tetrazolium-5-carboxanilide (XTT) in DMEM was added to the cells in the presence of 0.01% of N-methyl dibenzopyrazine methyl sulfate (PMS). After incubating for 4 h at 37°C, the plates were read in a spectrophotometer at 492 nm and 620 nm [22]. The 50% cytotoxic concentration (CC_50_) was calculated by a non-linear regression analysis of the dose-response curves.

### Yield-reduction assay

Monolayers of 5.5 x 10^6^ Huh-7 cells in 6-well plates were infected with YFV at an MOI of 0.1 for 1 h at 37°C. The cells were washed with PBS to remove residual viruses, and various concentrations of sofosbuvir, or ribavirin, in DMEM with 1% FBS were added. After 24 h, the cells were lysed, the cellular debris was cleared by centrifugation, and the virus titers in the supernatant were determined. A non-linear regression analysis of the dose-response curves was performed to calculate the concentration at which each drug inhibited the plaque-forming activity of YFV by 50% (EC_50_).

### Immunofluorescence analysis

Huh-7 and HepG2 cells were seeded on black 96-well microplates with clear bottom (Greiner Bio-One, Kremsmünster, Austria) and infected with YFV at an MOI of 1. After 1 hour, the viral inoculum was removed and cells were incubated with growth medium containing sofosbuvir, Ribavirin or AZT for 2 days. Cells were then fixed with 4% paraformaldehyde in PBS for 20 min at room temperature. The fixative was removed and cells monolayers were washed with PBS. Blocking of unspecific binding of the antibody and permeabilization were performed with 3% bovine serum albumin (BSA, Sigma Aldrich) and 0.1% Triton X-100 in PBS for 20 min at room temperature. SCICONS J2 antibody (Scicons, Hungary), which recognizes double-stranded RNA, was diluted 1:1000 in PBS and incubated for 1 h at room temperature. The primary antibody was removed and cell monolayer was washed twice with PBS. Secondary antibody Donkey anti-mouse IgG coupled to AlexaFluor488 fluorochrome (Thermo Fisher Scientific) was diluted 1:1000 in PBS and incubated for 40 min at room temperature. After washing cells with PBS, nucleus staining with DAPI diluted 1:10,000 in PBS was performed at room temperature for 10 min and then washed with PBS. Cells were imaged using a Nikon TE300 (Tokyo, Japan) inverted microscope coupled to a Leica DFC310FX camera (Leica Biosystems, Wetzlar, Germany). Images referent to AlexaFluor488 and DAPI signals were merged using the microscope software.

### Flow cytometry

Huh-7 and HepG2 cells were seeded in 6-well plates at density of 6 x10^4^ cells/well and 2.5 x 10^5^ cells/well, respectively. For infection, the growth medium was replaced by serum-free medium containing the virus at an MOI of 1. Mock-infected cells were incubated with conditioned medium from uninfected cells prepared exactly as performed for viral propagation. After 1 hour, inoculum was removed and replaced by growth medium containing either the vehicle or the antivirals at different concentrations and incubated for 48 h (for HepG2) or 72 h (for Huh-7) cells. After that, cells were harvested by treatment with a 0.25% trypsin solution. Cells were fixed with 4% paraformaldehyde (Sigma-Aldrich) in phosphate buffered saline (PBS) for 15 min at room temperature and washed with PBS. Cells were permeabilized with 0.1% Triton X-100 (Sigma Aldrich) in PBS, washed with PBS, and blocked with PBS with 5% FBS. Cells were incubated with 4G2, a pan-flavivirus antibody raised against the envelope E protein produced in 4G2-4-15 hybridoma cells (ATCC), diluted 1:10 in PBS with 5% FBS. Cells were labeled with donkey anti-mouse Alexa Fluor 488 antibody (Thermo Scientific,Waltham, MA, USA) diluted 1:1000 in PBS with 5% FBS, and were analyzed by flow cytometry in a BD Accuri C6 (Becton, Dickinson and Company, Franklin Lakes, NJ, USA) for YFV infection. The gate strategy to assure accuracy in the analysis is displayed as S1 Fig.

### Plaque forming assay

Virus titers were determined as PFU on Vero cells. Supernatants containing virus were serially diluted and incubated over confluent monolayers. After 1 h, cells were overlaid with semisolid medium, alpha-MEM (GIBCO) containing 1.4% carboxymethyl cellulose (Sigma-Aldrich) and 1% FBS (GIBCO). Cells were further incubated for 4 to 5 days. Cells were fixed in 4% formaldehyde and stained with 1% crystal violet in 20% ethanol for plaque visualization.

### Sequence comparisons

The sequences encoding the C-terminal portion of the RNA polymerase from Flaviviruses were acquired from the complete sequences deposited in GenBank. An alignment was performed using the ClustalW algorithm in the Mega 6.0 software. The sequences were analyzed by disparity index per site. Compared regions are displayed in the S1 Table.

### Comparative modeling

The amino acid sequence encoding YFV RNA polymerase (UniProtKB code: P03314) was obtained from the EXPASY proteomic portal [23] (http://ca.expasy.org/). The template search was performed using the Blast server (http://blast.ncbi.nlm.nih.gov/Blast.cgi) with the Protein Data Bank [24] (PDB; http://www.pdb.org/pdb/home/home.do) as the database and the default options. The T-COFFEE algorithm was used to generate the alignment between the amino acid sequences of the template proteins and YFV RNA polymerase. Subsequently, the construction of the YFV RNA polymerase complex was performed using MODELLER 9.19 software [25], which employs spatial restriction techniques based on the 3D-template structure. The preliminary model was refined in the same software, using three cycles of the default optimization protocol. The structural evaluation of the model was then performed using two independent algorithms in the SAVES server (http://nihserver.mbi.ucla.edu/SAVES_3/): PROCHECK software [26] (stereochemical quality analysis) and VERIFY 3D [27] (compatibility analysis between the 3D model and its own amino acid sequence by assigning a structural class based on its location and environment and by comparing the results with those of crystal structures).

### Animals

The procedures described in this study were in accordance with the ethical and animal experiments regulations of the Brazilian Government (Law 11794/2008), guidelines published at the Brazilian participant Institutions and National Institutes of Health guide for the care and use of laboratory animals. The study is reported in accordance with the ARRIVE guidelines for reporting experiments involving animals [28].

### Neonate mouse model

Swiss albino mice (*Mus musculus*) (pathogen-free) from the Oswaldo Cruz Foundation breeding unit (Instituto de Ciência e Tecnologia em Biomodelos; ICTB/Fiocruz) were used for these studies. The animals were kept at a constant temperature (25°C) with free access to chow and water in a 12-h light/dark cycle. The experimental laboratory received pregnant mice (at approximately the 14th gestational day) from the breeding unit. Pregnant mice were observed daily until delivery to accurately determine the postnatal day. We established a litter size of 10 animals for all experimental replicates. The Animal Welfare Committee of the Oswaldo Cruz Foundation (CEUA/FIOCRUZ) approved and covered (license number L-016/2016) the experiments in this study.

### Adult mouse model

In parallel, some experiments were conducted using type I interferon receptor deficient mice (A129^−/−^), SV129 background, obtained from Bioterio de Matrizes da Universidade de Sao Paulo (USP). Adult male and female A129^−/−^ mice were bred and kept at Immunopharmacology Laboratory of the Universidade Federal de Minas Gerais (UFMG) under specific pathogen-free conditions. Mice were kept at a constant temperature (25°C) with free access to chow and water in a 12-h light/dark cycle. Experimental protocol was approved by the Committee on Animal Ethics of the UFMG (CEUA/UFMG, Permit Protocol Number 84/2018).

#### Experimental infection and treatment

As proof-of-principle, sofosbuvir treatment was initially performed in three-day-old Swiss mice infected intraperitoneally with different doses of vaccinal virus. To monitor drug efficiency in a highly aggressive system, adult (7–9 week-old) A129^-/-^ (type I interferon receptor knockout) mice were infected with different inoculums of YFV by the intravenous (i.v.) route (tail vein). Both male and female mice were used in the experiments. Treatments with sofosbuvir were performed at 20 mg/kg/day administered intraperitoneally for Swiss newborn and orally (gavage) for A129^-/-^ mice. Regimen was administered daily beginning one day prior to infection or one day after infection until control group (animals infected and untreated) decease. Animals were monitored daily for survival and weight variation. Of note, both male and female mice were used in the experiments.

If necessary, euthanasia was performed to alleviate animal suffering. The criteria were the following: i) differences in weight between infected and control groups [> 50% for Swiss newborn (variation in weight gain) and > 20% for A129^-/-^ (variation in weight loss)], ii) ataxia, iii) loss of gait reflex, iv) absence of righting reflex within 60 seconds, and v) separation, with no feeding, of moribund offspring by the female adult mouse (for Swiss newborn mice).

#### Histopathological liver analysis

Liver samples from adult euthanized mice at day 3 post-YFV inoculation were obtained. Afterwards, they were immediately fixed in 10% buffered formalin for 24 h and embedded in paraffin. Tissue sections (4 mm thicknesses) were stained with hematoxylin and eosin (H&E) and evaluated under a microscope, Axioskop 40 (Carl Zeiss, Göttingen, Germany) adapted to a digital camera (PowerShot A620, Canon, Tokyo, Japan). Histopathology score was performed according to a set of custom designed criteria modified from evaluating cellular infiltration, hepatocyte swelling and degeneration and then added to reach a four-points score (0, absent; 1, slight; 2, moderate; 3, marked; and 4, severe) in each analysis [29]. For easy interpretation, the overall score was taken into account and all the parameters summed for a maximum possible score of 8 points. A total of two sections for each animal were examined and results were plotted as the media of damage values in each mouse.

#### Statistical analysis

All assays were performed and codified by one professional. Subsequently, a different professional analyzed the results before the identification of the experimental groups. This approach was used to keep the pharmacological assays blind. All experiments were carried out at least three independent times, including technical replicates in each assay. The dose-response curves used to calculate the EC_50_ and CC_50_ values were generated by Prism GraphPad software 7.0. The equations to fit the best curve were generated based on R^2^ values ≥ 0.9. Fisher's exact and ANOVA tests were also used, with *P* values <0.05 considered statistically significant. For flow cytometry and viral titer analysis, data were analyzed by ANOVA. When ANOVA revealed a significant effect, data were further analyzed with Dunnet post hoc test to correct for multiple comparisons and to determine specific group differences. The significance of survival curves was evaluated using the Log-rank (Mantel-Cox) test. *P* values of ≤0.05 were considered statistically significant.

## Results

### Prediction of the complex between Sofosbuvir triphosphate and YFV RNA polymerase using comparative modeling

We initially compared the disparities among regions encoding the RNA-dependent RNA polymerase (RDRP) region of the contemporary Flaviviruses DENV, ZIKV and YFV (Table 1). The YFV RNA polymerase shares a conserved domain for catalytic activity with the orthologous enzymes (S1 Table).

**Table 1:** Estimated disparities index per site between amino acid sequences encoding the RDRP domain peptide located at last 680 amino acids of C-terminal from contemporary flaviviruses.

			Distance comparisons				
Order	GenBank #	Agent	1	2	3	4	5
**1**	FJ654700.1	YFV					
**2**	KX197205.1	ZIKV	0.000				
**3**	AB189124.1	DENV1	0.000	0.002			
**4**	DQ672564.1	DENV2	0.000	0.000	0.056		
**5**	AY858046.2	DENV3	0.000	0.107	0.000	0.062	
**6**	GU289913.1	DENV4	0.000	0.042	0.000	0.011	0.000

Next, the crystal structures of DENV NS5 complexed with viral RNA (PDB code: 5DTO) [30],HCV NS5B in complex with Sofosbuvir diphosphate (PDB code: 4WTG) [31], and complexed to UTP (PDB code: 1GX6) [32] were selected and used in a comparative modeling procedure, covering 100% of the YFV RNA polymerase sequence considered here (residues Thr252-Ile878). These three template proteins represent orthologous viral RNA polymerases from the *Flaviviridae* family. Consequently, the resulting 3D model of YFV RNA polymerase in complex with Sofosbuvir triphosphate showed good structural quality.

**Fig 1:**
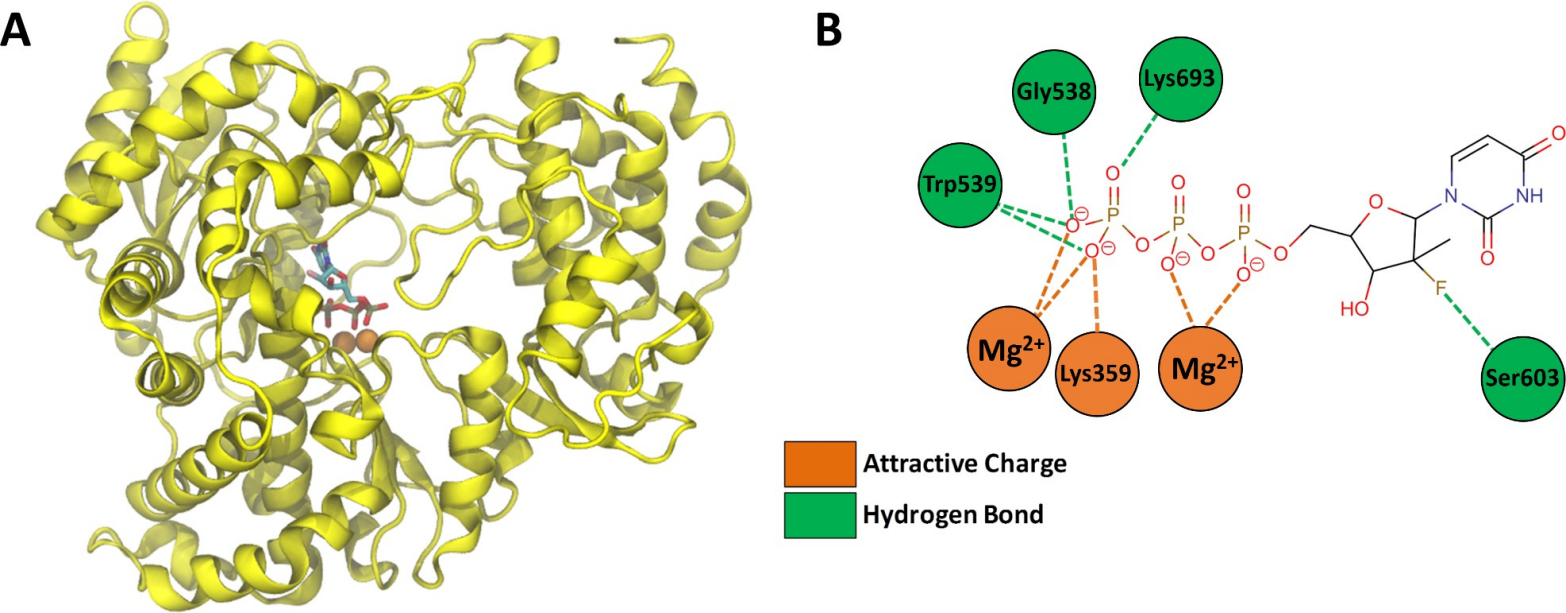
3D model of YFV RNA polymerase in complex with sofosbuvir triphosphate. (A) Cartoon representation of YFV RNA polymerase 3D model. The sofosbuvir triphosphate structure is represented by sticks and colored according to atom type (red, oxygen; blue, nitrogen; orange, carbon; yellow, phosphorus) and the two catalytic Mg^2+^ ions are shown as orange spheres. (B) Schematic representation of the interaction between amino acid residues of the enzyme and the sofosbuvir triphosphate structure, where the salt bridges are shown in orange and hydrogen bonds are in green.

The analysis of the YFV RNA polymerase model suggests that sofosbuvir triphosphate binds between the palm and the fingers regions, making hydrogen bonds with Gly538, Trp539, Ser603, and Lys 693 residues and salt bridge interactions with Lys359 and two Mg^2+^ ions. Interestingly, these interactions are all described as relevant for incorporation of natural ribonucleotides [31] (Fig 1). Therefore, these results motivate further testing in biologically relevant models to YFV replication.

#### Sofosbuvir inhibits YFV replication in different models of human hepatoma cells

**Fig 2:**
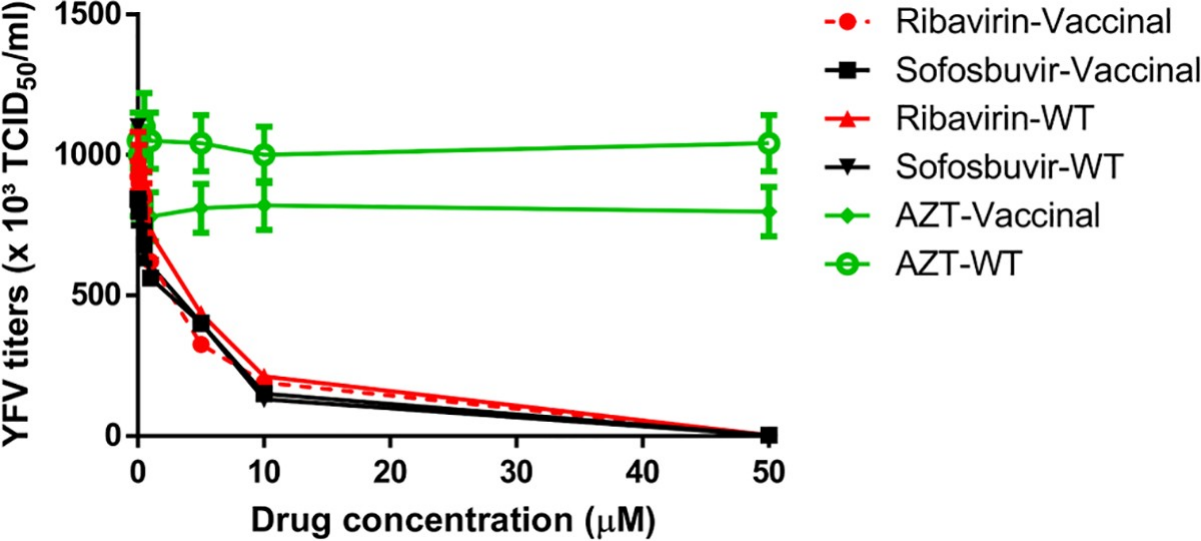
Pharmacology of sofosbuvir against YFV. Huh-7 were infected with YFV at an MOI of 0.1 and exposed to various concentrations of the antivirals for 24 h. Supernatants were harvested and titered in Vero cells by TCID_50_/mL. The data represent means ± SEM of five independent experiments.

YFV primarily replicates in the liver, where sofosbuvir is majorly converted from prodrug to its active metabolite [13–15]. Thus, we monitored YFV susceptibility to sofosbuvir using human hepatocellular carcinoma cells. YFV was yield in Huh-7 cells in the presence of sofosbuvir for 24 h, and then cell lysates were titered in Vero cells. Indeed, sofosbuvir inhibited both vaccine and WT strains of YFV replication similarly, in dose-dependent manner (Fig 2). As a positive control to inhibit virus replication, we used ribavirin, a broad spectrum antiviral which was previously shown to inhibit YFV replication *in vitro* and *in vivo* [33–35] (Fig 2). Sofosbuvir’s and ribavirin’s potencies over YFV were quite comparable, with EC_50_ values varying 12% (Table 2). Of note, AZT, used as a negative control, did not affect virus replication (Fig 2).

**Table 2:** Pharmacological parameters related to antiviral activity of sofosbuvir and ribavirin against YFV.

Drugs	EC_50_ (μM)		CC_50_ (μM)	SI*	
	Vaccinal	WT		Vaccinal	WT
Sofosbuvir	4.8 ± 0.2	4.2 ± 0.2	381 ± 25	80	90
Ribavirin	3.9 ± 0.3	4.9 ± 0.3	284 ± 12	72	58

*Selectivity index (SI) = CC_50_/EC_50_

Since sofosbuvir is around 25% less cytotoxic than ribavirin, its selectivity index (SI; ratio between EC_50_ and CC_50_) was also higher (Table 2). Sofosbuvir displayed improved SI values (when compared to ribavirin) by 10 and 50% with respect to the vaccine and WT strains, respectively (Table 2).

**Fig 3:**
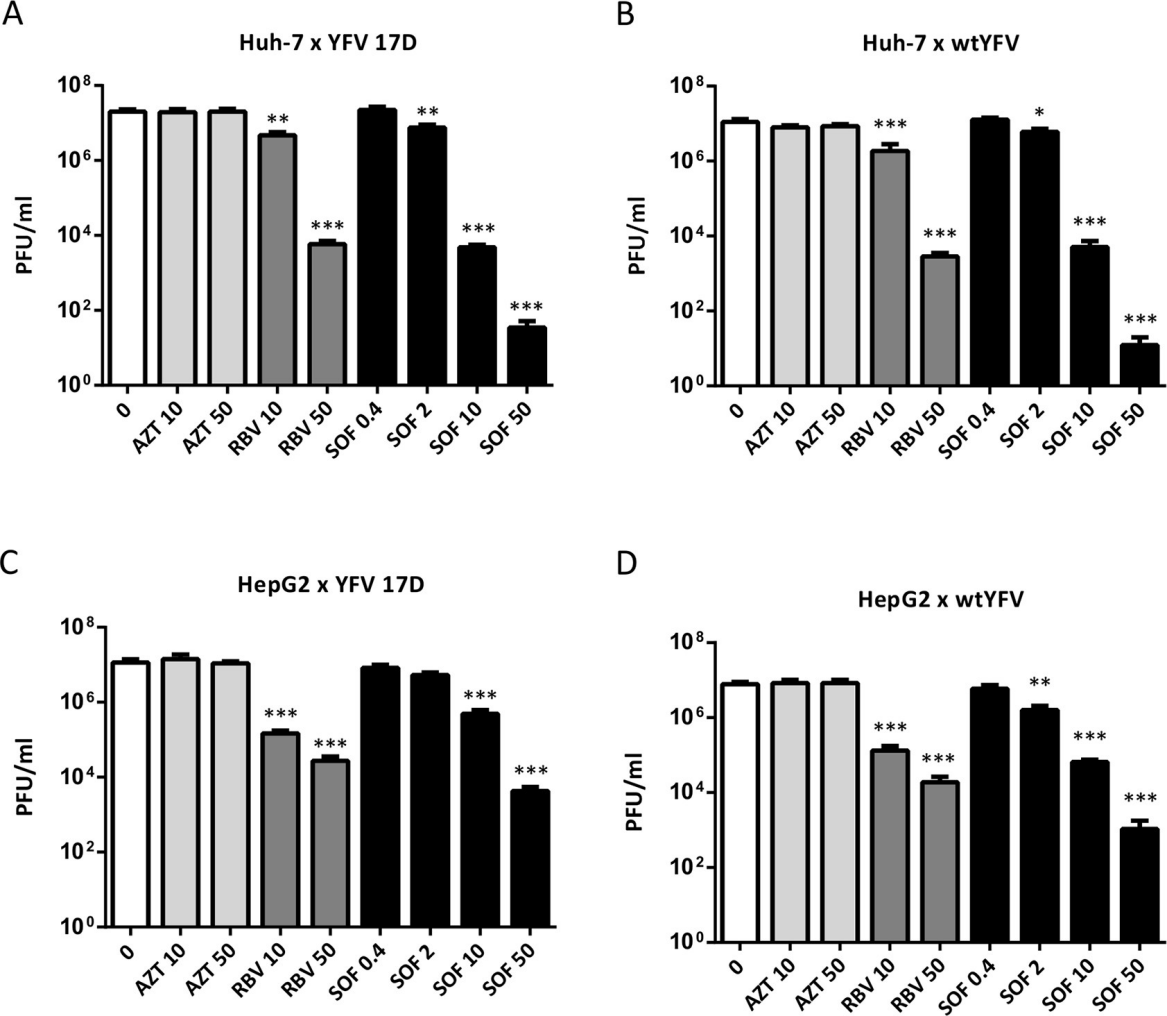
Sofosbuvir reduces the production of YFV viral particles. Huh-7 cells were infected with vaccine strain YFV17D (A) or WT-YFV (B) at an MOI of 1. HepG2 cells were infected with vaccine strain YFV17D (C) or WT-YFV (D) at an MOI of 1. YFV-infected were exposed to the indicated concentrations of AZT, ribavirin, or sofosbuvir. Supernatants were analyzed by plaque assay to determine viral titers. Data represent mean ± SEM of at least four independent experiments. Comparison between untreated and treated YFV-infected cells was performed using ANOVA followed by Dunnet post hoc test. Statistical significance compared to untreated YFV-infected cells is indicated by asterisks (* P < 0.05, ** P < 0.01 and *** P <0.001).

We further expanded whether YFV susceptibility to sofosbuvir was similar in different lineages of hepatoma cells, Huh-7 and HepG2. To do so, titers were determined by PFU, for more accurate comparisons, with respect to differences in levels of virus replication. Consistently, sofosbuvir treatment decreased the production of infectious virus particles (Fig 3) in a dose-dependent manner in Huh-7 (Fig 3A and 3B) and HepG2 cells (Fig 3C and 3D). Ribavirin also inhibited viral replication, whereas AZT was ineffective (Fig 3). Sofosbuvir at doses of 10 and 50 μM inhibited, respectively, 3- and 5-log_10_ the production of infectious particles by infected Huh-7 cells (Fig 3A and 3B). Sofosbuvir was at least 100 times more effective than ribavirin at inhibiting YFV infectivity (regardless of strain) in infected Huh-7 cells (Fig 3A and 3B). YFV susceptibility to the tested antiviral drugs in HepG2 cells was lesser when compared to Huh-7 cell (Fig 3A, 3B, 3C and 3D). Sofosbuvir treatment reduced the production of infectious YFV in HepG2 cells by 1- and 2-log_10_ at doses of 10 and 50 μM, respectively (Fig 3C and 3D). In HepG2 cells, ribavirin’s and sofosbuvir’s effects over virus replication were comparable (Fig 3C and 3D).

**Fig 4:**
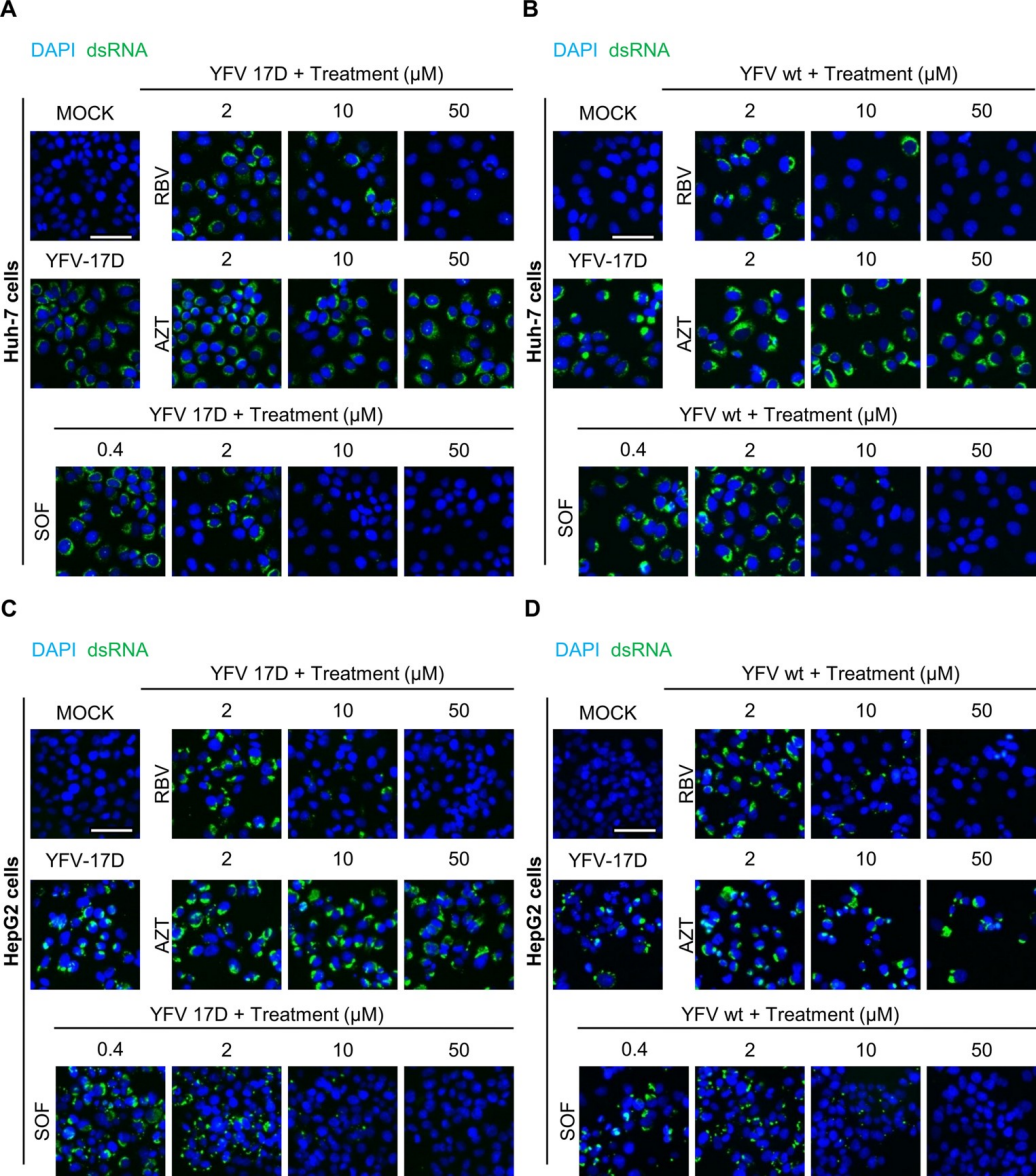
Sofosbuvir inhibits viral dsRNA, an intermediate in viral genome replication. Huh-7 cells were infected with either vaccine strain, YFV-17D (A) or WT-YFV (B); HepG2 cells were infected with YFV-17D (C) or WT-YFV (D). Infected cells were treated with AZT, ribavirin (RBV), or sofosbuvir (SOF) at the indicated concentrations for 48 h. Viral replication was analyzed by immunofluorescence using J2 antibody to detect viral replication intermediate dsRNA (green) and DAPI to stain cell nuclei (blue). White scale bars indicate 50 μm. Images are representative of four independent experiments.

Knowing the pharmacological activity and putative target, we used a cell-based assay to demonstrate that sofosbuvir inhibits YFV RNA polymerase. During YFV replication, an anti-genomic negative-sense RNA strand is synthetized, creating an intermediate double-stranded (ds) RNA with the positive-sense virus genome. Viral genome replication was thus assessed by immunodetection of dsRNA. HepG2 and Huh-7 hepatoma cells lines were infected with an MOI of 1 and treated with sofosbuvir (0.4 to 50 μM) for 48 h. Ribavirin and AZT were used as positive and negative controls, respectively. Sofosbuvir reduced the genome replication of both vaccine and WT strains of YFV in Huh-7 and HepG2 cells in a dose dependent manner (Fig 4). Again, YFV replication was more susceptible to sofosbuvir in Huh-7 (Fig 4A and 4B) than HepG2 (Fig 4C and 4D) cells. Although ribavirin showed activity both against YFV 17D and WT YFV, full inhibition was only achieved at higher concentrations (when compared to sofosbuvir) (Fig 4). In Huh-7 cells treated with 10 μM of the drugs, we observe less foci of dsRNA in sofosbuvir- than in ribavirin-treated cells (Fig 4A and 4B). AZT was inefficient in blocking either vaccine or WT YFV replication at the tested concentrations (Fig 4).

**Fig 5:**
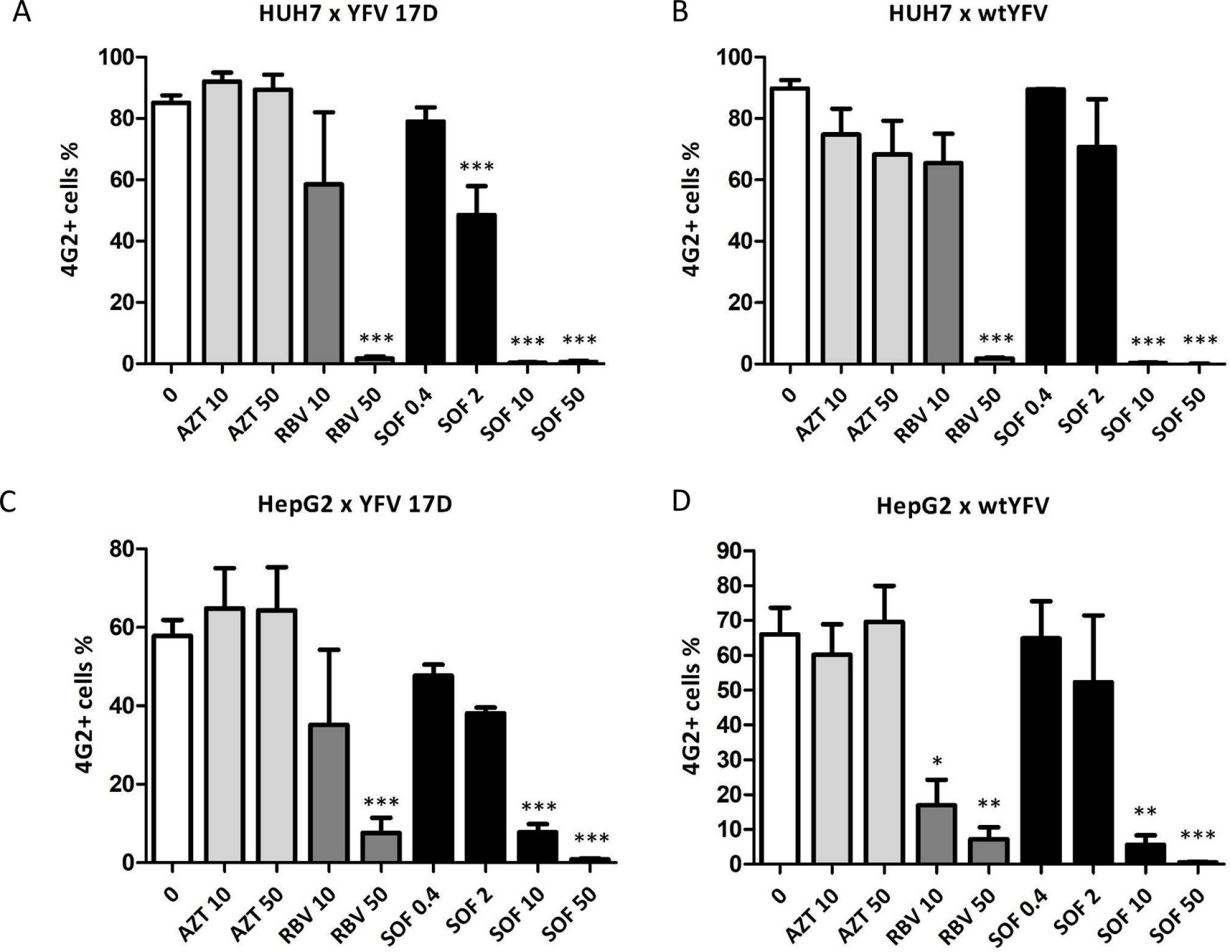
Sofosbuvir reduces the number of YFV-infected cells. Huh-7 cells were infected with vaccine strain YFV17D (A) or wild-type YFV (B) at an MOI of 1. HepG2 cells were infected with vaccine strain YFV17D (C) or wild-type YFV (D) at an MOI of 1. YFV-infected cells were exposed to the indicated concentrations of AZT, ribavirin, or sofosbuvir. Anti-pan-flavivirus antibody was used for flow cytometry analysis. The data represent mean ± SEM of at least three independent experiments. Differences between untreated and treated YFV-infected cells were assessed using ANOVA followed by Dunnet post hoc test. Statistical significance compared to untreated YFV-infected cells is indicated by asterisks (* P < 0.05, ** P < 0.01 and *** P <0.001).

We next determined antigen production by flow cytometry using the 4G2 antibody, a pan-flavivirus antibody raised against the envelope protein (Fig 5 and S2 Fig). Huh-7 cells were infected with vaccine (Fig 5A) or WT (Fig 5B) YFV strain at an MOI of 1 and then treated with ribavirin or AZT at 10 and 50 μM or with sofosbuvir in concentrations ranging from 0.4 to 50 μM for 72 h. Flow cytometry analysis showed a pronounced reduction in the number of YFV-infected Huh-7 cells treated with 50 μM ribavirin and 10–50 μM sofosbuvir compared to untreated YFV-infected cells (Fig 5A and 5B). AZT treatment did not present a significant anti-YFV effect. Untreated WT YFV-infected cells presented with 89.75% of 4G2+ cells whereas sofosbuvir treatment at 10 and 50 μM resulted in 0.36 and 0.06% of antigen positive cells, respectively. Treatment with 10 and 50 μM ribavirin led to 65.43 and 1.79% of YFV-infected cells, indicating that sofosbuvir was more efficient than ribavirin in reducing the number of infected cells. HepG2 cells were also infected with vaccine (Fig 5C) or WT (Fig 5D) YFV and treated with AZT, ribavirin, or sofosbuvir for 48 h and analyzed by flow cytometry. Similar to the results observed in Huh-7, ribavirin and sofosbuvir treatment led to a reduction in the number of YFV-infected HepG2 cells. Noteworthy, sofosbuvir and ribavirin-induced reduction of YFV-infected cells was more pronounced in Huh-7 cells than in HepG2 cells (Fig 5). This result is consistent with our observations from immunodetection of viral dsRNA (Fig 4). Sofosbuvir treatment reduced the number of YFV-infected cells in a dose-dependent manner, both in Huh-7 and HepG2 cells (Fig 5). We did not observe differences in sofosbuvir susceptibility between vaccine and WT YFV strain, with respect to antigenic production.

Altogether, sofosbuvir demonstrated higher potency in the reduction of viral genome replication, protein synthesis, and infectious viral particle production than ribavirin in both hepatoma cell lines tested. These results indicate that sofosbuvir is a good candidate against YFV and deserving of further *in vivo* testing.

#### Sofosbuvir enhances survival of YFV-infected mice

**Fig 6:**
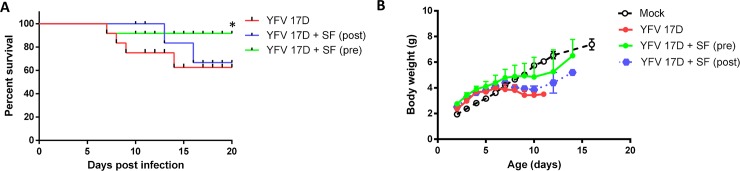
Early/Prophylactic treatment with sofosbuvir increases survival of YFV-infected mice. Three-day-old Swiss mice were infected with YFV-17D (1 x 10^4^ PFU) and treated with sofosbuvir either 1 day before (pre) or after (late) infection. Survival (A) and weight variation (B) were assessed during the course of treatment. Survival was statistically assessed by Log-rank (Mentel-Cox) test. Differences in weight are displayed as the means ± SEM. At least three independent experiments were performed with 10 mice/group. * P < 0.05.

To test sofosbuvir *in vivo*, we used the dose of 20 mg/kg/day in newborn Swiss outbred mice. This dose is consistent with its pre-clinical/clinical studies for drug approval [15]. Since *in vitro* experiments had shown that vaccine and WT strains of YFV are equally susceptible to sofosbuvir, we initially used the vaccine strain because it required easier handling and biosafety containment. Three-days-old Swiss mice were infected intraperitoneally with 1.0 x10^4^ PFU of vaccine virus. These animals began to receive treatment 1 day prior to infection (pre-treatment) or 1 day post-infection (late-treatment). Sofosbuvir significantly enhanced the survival of YFV-infected mice who received pre-treatment, pointing out to prophylactic activity and a possible narrow time frame for antiviral intervention (Fig 6A). Variations in body weight among groups were marginal (Fig 6B). Despite the fact that mortality starts to occur only 7 days after infection, late-treatment did not statistically increase animal survival (Fig 6A).

**Fig 7:**
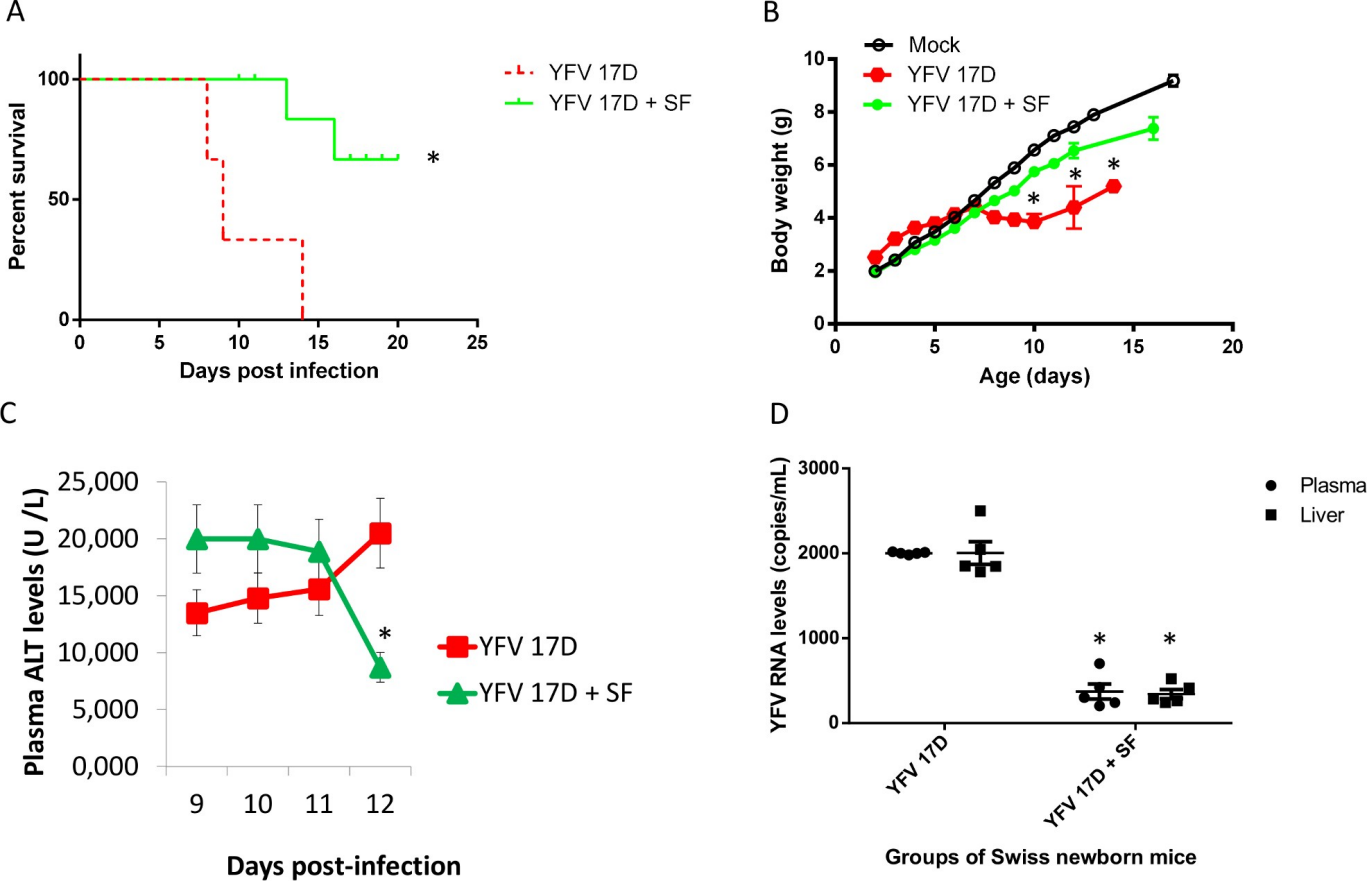
Pre-treatment with sofosbuvir increases survival and inhibits weight loss of YFV-infected mice. Three-day-old Swiss mice were pre-treated with sofosbuvir beginning 1 day before infection with YFV 17D (1 x 10^6^ PFU). Survival (A) and weight variation (B) were assessed during the course of treatment. Survival was statistically assessed by Log-rank (Mentel-Cox) test. Differences in weight are displayed as the means ± SEM. Three-independent experiments were performed with 10 mice/group. Throughout the course of the experiment, a subset of animals (3 mice/group) were euthanized to monitor plasma ALT (C) and viral RNA levels (D). Data are presented as means ± SEM. For panels B, C and D, two-way ANOVA for each day was used to assess the significance * P < 0.05.

Next, we repeated the same model of three-days-old Swiss mouse infection, but with a higher dose of vaccine virus (1 x 10^6^ PFU) to achieve higher mortality. Since late-treatment did not previously enhance survival in this model, the efficacy of sofosbuvir was accessed only with the pre-treatment condition. Sofosbuvir protected pre-treated mice from YFV-induced mortality (Fig 7A). Infected mice died within two weeks after infection, whereas 70% of sofosbuvir pre-treated YFV-infected animals survived to lethal inoculum (Fig 7A). We also evaluated the weight gain during the time course of our experiment in mice. YFV-infected animals had reduced postnatal development, whereas sofosbuvir-treated YFV-infected mice gained weight almost as much as the uninfected controls (Fig 7B). We monitored through the course of infection the levels of alanine aminotransferase (ALT), an important biomarker of liver integrity. At day 12 post-infection, when untreated animals experience the highest mortality (Fig 7A), ALT levels became two-times higher in the infected/untreated mice than in those treated with sofosbuvir (Fig 7C). Consistent with the increase in this biomarker of liver injury, untreated animals displayed viral replication almost three-times higher in blood plasma and liver than treated mice (Fig 7D).

**Fig 8:**
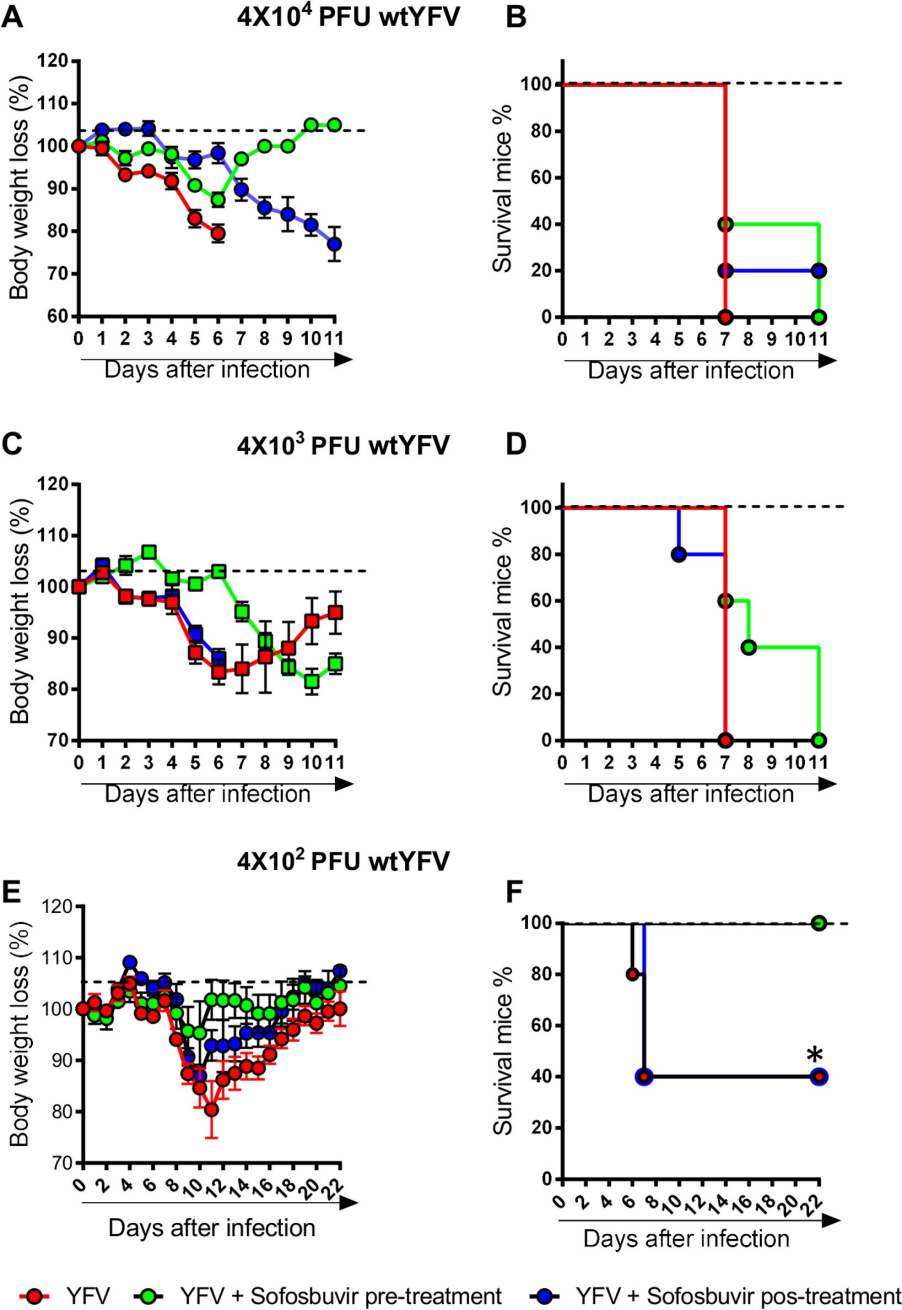
Treatment with sofosbuvir increases survival and inhibits weight loss of adult YFV-infected A129^-/-^ mice. 7–9 week-old A129^-/-^ mice were inoculated with 4x10^4^, 4x10^3^ or 4x10^2^ PFU of a WT strain of YFV i.v. A, C and E) Body weight was measured daily until day 14^th^ and expressed as % of body weight loss from day 0 before YFV inoculation. B, D and F) Survival rates were analysed every 12 hours and expressed as % of survival mice until day 14^th^ post-YFV inoculation. Dashed lines are representative of MOCK-infected group. Survival was statistically assessed by Log-rank (Mentel-Cox) test. Differences in weight are displayed as the means ± SEM, and two-way ANOVA for each day was used to assess the significance. Three independent experiments were performed with at least four mice/group. * P < 0.05.

Although these initial *in vivo* results are exciting, documented information of sofosbuvir protection in a highly virulent *in vivo* system is noteworthy. Mice with impaired innate immune response, due to the lack of type I interferon receptor (A129^-/-^), were infected with WT YFV and treated with sofosbuvir at 20 mg/kg/day, again beginning one day before (pre-treatment) or after (post-treatment) infection. Results show that inoculation with 4 x 10^4^ and 4 x 10^3^ PFUs caused 100% mortality within one week along with massive loss in body weight (Fig 8). At these high virus inputs, pre-treatment with sofosbuvir enhanced the mean time of survival by 57%, although mortality was the final outcome for all mice (Fig 8A and 8B). At a virus dose able to cause 50% of mortality, 4 x 10^2^ PFU, pre-treatment with sofosbuvir enhanced survival significantly (Fig 8C).

**Fig 9:**
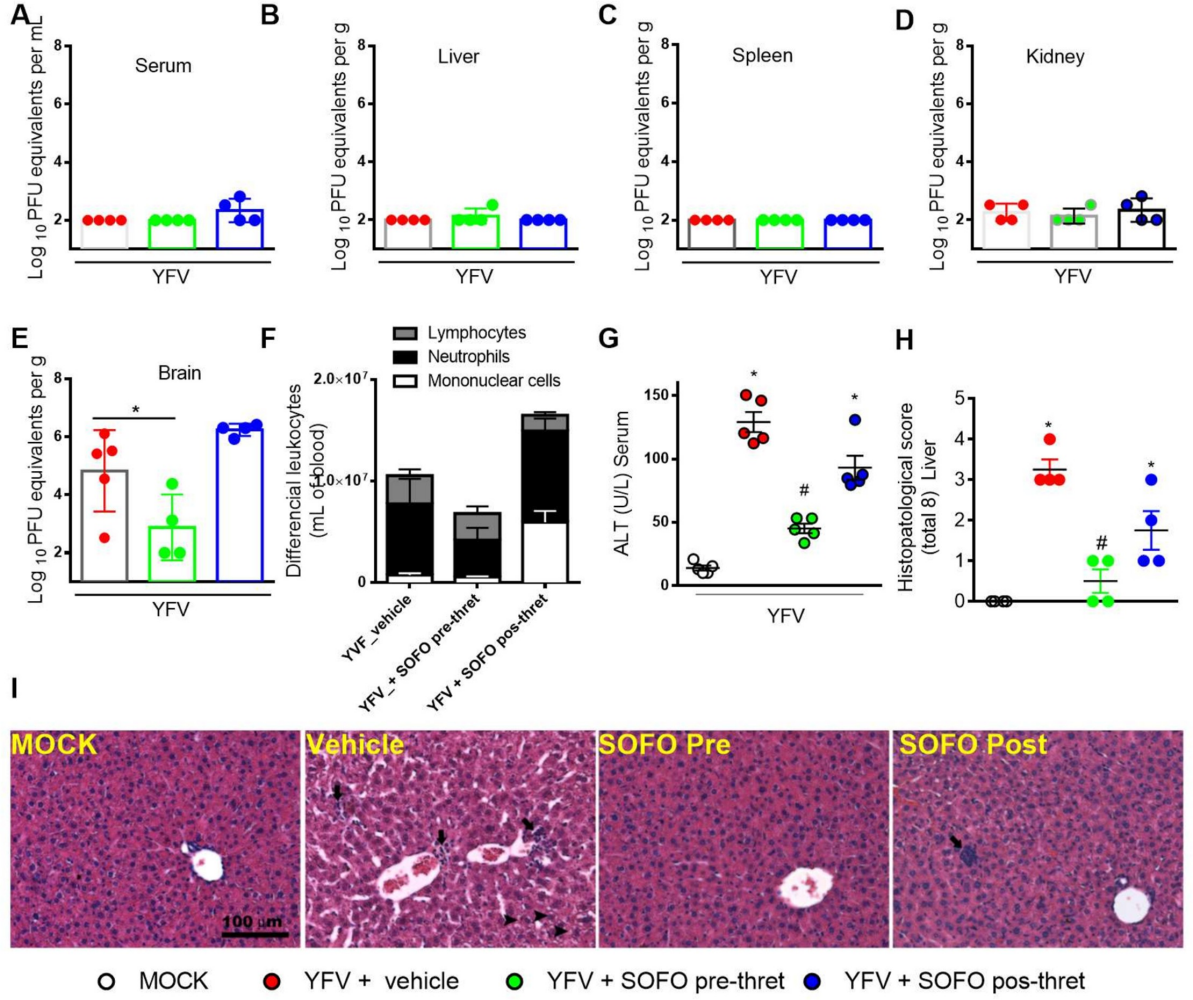
Treatment with Sofosbuvir ameliorates disease outcomes of adult YFV-infected A129^-/-^ mice. A129^-/-^ mice were infected with 4x10^2^ PFU of low passage clinical isolate of YFV by intravenous route. A-E) Viral loads from serum (A), liver (B), spleen (C), kidneys (D), and brain (E) assessed by plaque assay. Results are shown as median f Log_10_ PFU equivalents per mL or g. F) Total and differential cell counts in blood were represented as number of differential cell counts (leukocytes, mononuclear cells and neutrophils) normalized to % of total cells counts. G) Hepatic transaminase levels of ALT were measured in serum of MOCK and YFV-infected mice at days 3 and 6 p.i. Results are shown as ALT (U/L) of serum. H) Histopathological score of liver tissue sections. I) Representative of hepatic damage and Hematoxylin & Eosin staining of liver sections of control and YFV-infected mice three days after infection (Scale Bar—100 μm). The images presented are representative animals on the third day of infection. Four animals per group were assayed. * for p<0.05 when compared to MOCK. # for p< 0.05 when compared to YFV.

Taking this last condition as reference, infectious viral titers were measured in different organs, along with serum ALT levels, liver histopathology, and white cell counts. WT YFV replicated in different anatomical compartments of the A129^-/-^, like peripheral blood, liver, spleen and kidney (Fig 9A–9D). In parallel to viscerotropic replication, animals rapidly progress to high virus titers in the brain (Fig 9E). Pre-treatment with sofosbuvir reduced the virus levels in the brain of the infected mice by 2-log (Fig 9E), along with leucocytosis (Fig 9F). Hepatic condition was improved in sofosbuvir-treated mice, regardless of the regimen, indicated by measuring ALT levels (Fig 9G) and liver injury (Fig 9H and 9I).

Although pre-treatment was to be better than late-treatment to enhance survival, hepatic histology and ALT levels show that benefits from late treatment may exist (Fig 9G–9I). Histopathological analysis revealed mild alterations in the liver of vehicle-treated mice, including discrete and focal neutrophil inflammatory infiltrate and hepatocyte degeneration. Interestingly, pre-treatment with sofosbuvir abrogated such alterations while post-treatment partially reverted the observed liver lesions. This last result show that, although limited in efficacy, late treatment may be better than leaving the animals untreated. Our in *vivo* data reinforces the repurposing of sofosbuvir towards YF, with limited timing opportunity for intervention.

## Discussion

Brazil has been challenged in recent years by the (re)emergence of arboviruses. Massive cases of microcephaly associated with ZIKV circulation were registered in 2015/16 [36]. Two different genotypes of chikungunya virus (CHIKV) started to co-circulate from 2014 onward [37, 38]. The four DENV serotypes are hyperendemic throughout the country [39]. More recently, YFV activity increased, affecting both non-human primates and humans without constituted immunity [3, 6]. These facts clearly demonstrate that strategies of vector control failed and highlights the necessity of alternative strategies to control or even mitigate the diseases provoked by the arboviruses.

In the context of this article, re-emergence of YFV also points out that poor vaccination coverage left human population living in or entering the ecotone susceptible to this virus. Unvaccinated individuals may quickly progress to severe YF, with hepatic and even neurological impairment [1]. In addition, due to massive YF vaccine campaigns, a fair amount of vaccine-related severe adverse events may be expected. Thus, finding antiviral drugs to treat YFV-infected individuals is critical for medical intervention over cases provoked by WT or even vaccine strains.

Several groups demonstrated that the clinically approved anti-HCV drug sofosbuvir is endowed with antiviral activity towards other flaviviruses, such as ZIKV [10, 40] and DENV [12]. Considering that these agents belong to the same family, it was plausible to test sofosbuvir against YFV. Indeed, we observed that sofosbuvir targets the YFV RNA polymerase *in silico* and inhibits YFV RNA replication and infectious virus production. The *in vitro* antiviral results display that sofosbuvir’s EC_50_ towards YFV is in micromolar range and comparable to what has been described for ZIKV and DENV [10, 12, 40].

Our data showed that sofosbuvir inhibits YFV in hepatoma cell lines. This is particularly relevant since YFV targets hepatocytes and the liver is the most affected organ in YF [41]. The degree of liver damage measured by elevated aminotransferase levels and jaundice is associated with higher mortality [42]. Our Swiss mouse neonate model of virus infection reproduced the deleterious association among increased ALT, viral loads, and mortality. By inhibiting virus replication, sofosbuvir attacked this deleterious loop towards the liver protection and enhancement of the survival. In humans, massive apoptosis and necrosis of hepatocytes are reported in fatal cases [43]. Additionally, impaired synthesis of clotting factors caused by YFV-induced liver injury is key to the pathogenesis of haemorrhagic manifestations in severe YF [44]. Experimental cases of liver transplant after YFV infection have been conducted during recent outbreak in Brazil [4, 45, 46] with 50% survival rate. We envision that sofosbuvir may improve liver function in YF patients, and hopefully enhance the survival rates of transplanted individuals.

In the highly virulent infection mouse model, A129^-/-^ mice infected with WT YFV, sofosbuvir also diminished virus replication in the brain and improved liver condition. Both viscerotropic and neurological replication injuries are hallmarks of YFV pathogenesis [8, 47, 48]. Our data point to a possible benefit of early treatment with sofosbuvir for patients that may progress to complications at late stages in the natural history of infection. Interestingly, although sofosbuvir was more efficient when used prophylactically, it was able to improve the hepatic condition of the infected animals receiving a post-infection treatment.

Sofosbuvir reduced the YFV-induced mortality and lack of weight gain in mice models. Considering our data and the safety history of sofosbuvir in hepatitis, it is not a hyperbolic conclusion to consider sofosbuvir in clinical use for YF infection in humans. Primarily, sofosbuvir could be worthwhile for acutely infected individuals and those displaying neurotropic and viscerotropic diseases provoked by virus replication. Since vaccine is not recommended for special groups of individuals at higher risk of severe adverse events, such as elderly and those with immunodeficiencies, sofosbuvir could be used prophylactically.

Most commonly, we used ribavirin, a pan-antiviral drug with anti-YFV activity demonstrated both *in vitro* and *in vivo* [33–35], as a positive control to inhibit YFV replication and to compare with sofosbuvir. Our *in vitro* data showed that sofosbuvir was more efficient than ribavirin in reducing viral genome replication, number of infected cells, and production of infectious viral particles in Huh-7 cells. Moreover, we thus understand that sofosbuvir possesses advantages over ribavirin in terms of safety and efficacy. Ribavirin is more toxic than sofosbuvir, especially for critically ill hepatic and renal patients [49], such as those with YF. According to FDA categorization of risk during pregnancy; sofosbuvir is class B (drugs with the second to lowest risk of causing malformations), whereas ribavirin is class X (forbidden, even for men having intimate contact with women at gestational age). Thus, ribavirin would have a more limited scope of use. Indeed, when ribavirin was used in a clinical trial against a flavivirus, the Japanese encephalitis virus, it failed to be effective [50].

Although our results are translational, it is important to cite that further studies are necessary to precisely determine sofosbuvir’s mechanism(s) of action towards YFV life cycle and the dose adjustment to treat patients with YF. YFV RNA polymerase is the likely target, based on docking onto this enzyme and reduction of viral dsRNA levels. As with other RNA polymerases from positive-sense RNA viruses, well-conserved motifs are found the YFV and HCV RNA polymerase, such as D-x(4,5)-D and GDD, which are spatially juxtaposed, wherein Asp binds Mg^2+^ and Asn selects ribonucleotide triphosphates over dNTPs, determining RNA synthesis [9]. It would not be surprising to see sofosbuvir triphosphate bound to these critical residues for catalysis, because even other positive-sense RNA virus beyond members of the Flaviviridae family are susceptible to sofosbuvir [51]. Sofosbuvir inhibits HCV RNA polymerase as chain terminator [31]. Nevertheless, this drug is considered a non-obligate chain terminator, due to the presence of 3’-OH moiety. Indeed, sofosbuvir inhibited ZIKV replication by targeting its RNA polymerase directly and provoking A-to-G mutation in the virus genome [40]. Whether sofosbuvir acts directly on YFV RNA polymerase, induces an error-prone virus replication by its incorporation in the virus genome or inhibition inosine monophosphate dehydrogenase and/or independent mechanisms–it remains to be elucidated. We also observed that sofosbuvir inhibits 100- to1000-times more potently HCV than YFV production/replication [13, 31, 52]. These differences in potencies need to be interpreted in light of the sofosbuvir’s concentrations found in anatomical compartments and its long-half life [16], which may already be enough to compensate the limitation in the *in vitro* pharmacological potency. Sofosbuvir’s chemical structure allows its vectorization to the liver, where it is found at 77 μM when patients are treated with the reference dose of 400 mg/day [53]. YFV production was reduced up to 3-log_10_ when hepatoma cells where treated with sofosbuvir at 50 μM. Since sofosbuvir has been used clinically at doses up to 1200 mg/day [16], it is possible to have enough sofosbuvir in the liver to inhibit or reduce YFV replication in humans remains. Only clinical trials will reveal the best regimen and posology for sofosbuvir towards YF. In the our mouse models, sofosbuvir efficacy was observed at the reference pre-clinical dose previously studied for HCV, 20 mg/kg/day [15].

The results described here demonstrate for the first time the antiviral activity of sofosbuvir to YFV, which caused a recent outbreak in Brazil, providing primary scientific evidence for a new potential use of a clinically approved antiviral drug and reinforcing that its chemical structure may be used to generate selective anti-YFV specific drugs.

## Supporting information

S1 Fig Gate strategy from flow cytometry analysis. Flow cytometry events were gated on a dot plot FSC-A x FSC-H to exclude doublets (A-C). Cells were identified on FSC-A x SSC-A dotplots and gated to eliminate debris from analysis (D-F) and then evaluate percentage of 4G2-positive cells (G-I). Panels are representative of five independent experiments.
(PDF)

S2 Fig Dot plots from flow cytometry analysis. 4G2 positive cells quantified from Huh-7 (A-E) and HepG2 (F-J) cells infected with YFV and treated with sofosbuvir at indicated concentrations. Representative of at least five independent experiments.
(PDF)

S1 Table Alignment of RNA polymerases from members of the Flaviviridae family. C-terminal region of the RNA polymerase from Zika, Dengue, hepatitis C and yellow fever viruses. Conserved amino acid residues are highlighted in yellow. Critical amino acid residues are highlighted in red.
(PDF)
