# Supplementary material for: Yellow fever virus is susceptible to sofosbuvir both in vitro and in vivo
Source: PLoS Negl Trop Dis. 2019 Jan 30;13(1):e0007072. doi: 10.1371/journal.pntd.0007072 (PMC6375661; doi:10.1371/journal.pntd.0007072)
Supplement: S1 Fig — Flow cytometry events were gated on a dot plot FSC-A x FSC-H to exclude doublets (A-C). Cells were identified on FSC-A x SSC-A dotplots and gated to eliminate debris from analysis (D-F) and then evaluate percentage of 4G2-positive cells (G-I). Panels are representative of five independent experiments. (PDF) [file pntd.0007072.s001.pdf]

Figure S1

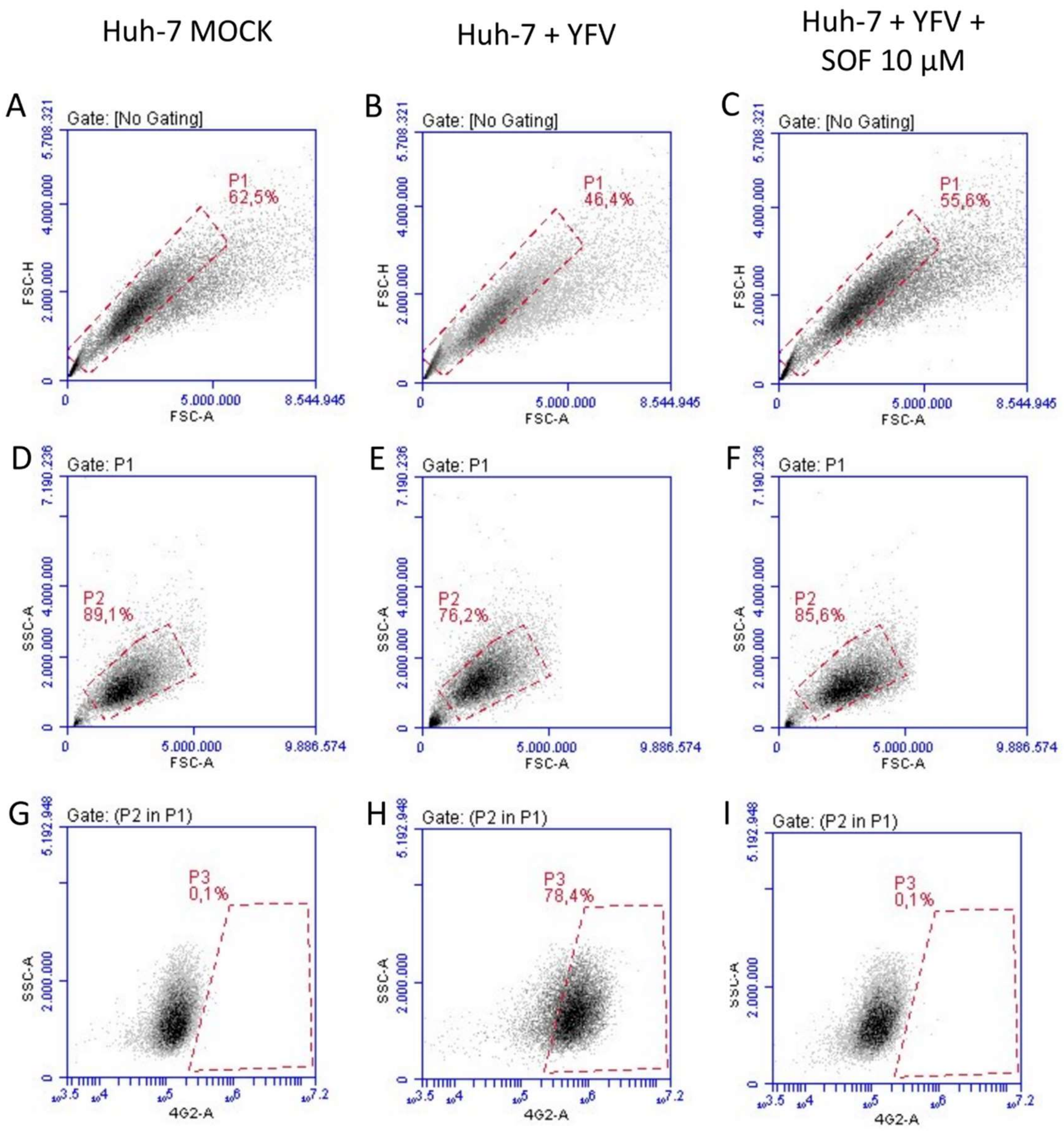

Figure S1. Gate strategy from flow cytometry analysis. Flow cytometry events were gated on a dot plot FSC-A x FSC-H to exclude doublets (A-C). Cells were identified on FSC-A x SSC-A dotplots and gated to eliminate debris from analysis (D-F) and then evaluate percentage of 4G2-positive cells (G-I). Panels are representative of over five independent experiments.
