# Supplementary material for: Yellow fever virus is susceptible to sofosbuvir both in vitro and in vivo
Source: PLoS Negl Trop Dis. 2019 Jan 30;13(1):e0007072. doi: 10.1371/journal.pntd.0007072 (PMC6375661; doi:10.1371/journal.pntd.0007072)
Supplement: S2 Fig — 4G2 positive cells quantified from Huh-7 (A-E) and HepG2 (F-J) cells infected with YFV and treated with sofosbuvir at indicated concentrations. Representative of at least five independent experiments. (PDF) [file pntd.0007072.s002.pdf]

Figure S2

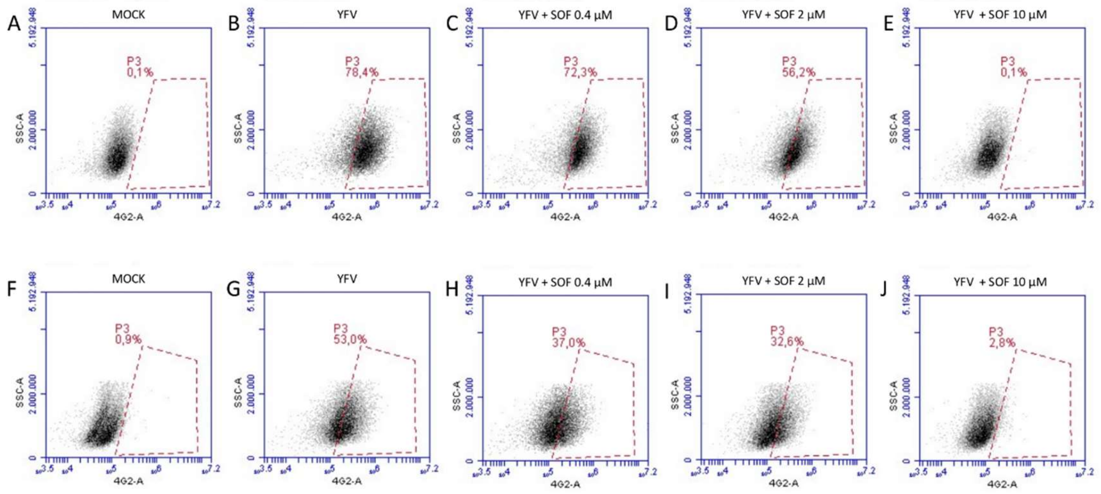

Figure S2. Dot plots from flow cytometry analysis. 4G2 positive cells quantified from Huh-7 (A-E) and HepG2 (F-J) cells infected with YFV and treated with sofosbuvir at indicated concentrations. Representative of at least five independente experiments.
