# Supplementary material for: Yellow fever virus is susceptible to sofosbuvir both in vitro and in vivo
Source: PLoS Negl Trop Dis. 2019 Jan 30;13(1):e0007072. doi: 10.1371/journal.pntd.0007072 (PMC6375661; doi:10.1371/journal.pntd.0007072)
Supplement: S1 Table — C-terminal region of the RNA polymerase from Zika, Dengue, hepatitis C and yellow fever viruses. Conserved amino acid residues are highlighted in yellow. Critical amino acid residues are highlighted in red. (PDF) [file pntd.0007072.s003.pdf]

Table S1 - Alignment of RNA polymerases from members of the Flaviviridae family

Domain: Data

Sequence	Alignment	Position
Zika virus strain ZikaSPH2015 complete genome	KYEEDVNLGSGTRAVVSCAEAPNMKIIGNRIERIRSEHAETWFFDENHPYRTWAYHGSYEAPTQGSASSLINGVVRLLSK	[80]
Dengue virus 2 isolate DENV-2/PK complete genome	TYEPDVDLGSGTRNIGIESETPNLDIIGKRIEKIKQEHETSWHYDQDHPYKTWAYHGSYETKQTGSASSVVNGVVRLLTK	[80]
Hepatitis C virus subtype 2a isolate PR63cc complete genome	--------GALITPCSPEEEKLPINPLSNSLLR---------------------YHNKVYCTTSKSASQRAKKVT-----	[80]
Hepatitis C virus subtype 1b isolate HCV-1b/US/BID-V148/2004 complete genome	--------GALITPCAAEESKLPINALSNSLLR---------------------HHNMVYATTSRSASQRQKKVT-----	[80]
Hepatitis C virus subtype 3a isolate ILBSRAS UG polyprotein gene complete cds	--------GALITPCSAEEEKLPISPLSNSLLR---------------------HHNLVYSTSSRSASQRQKKVT-----	[80]
Hepatitis C virus subtype 4a isolate 25 polyprotein gene complete cds	--------GALVTPCAAEESKLPISPLSNSLLR---------------------HHNMVYATTTRSAVTRQK?VT-----	[80]
AB189124.1 Dengue virus 2 genomic RNA complete genome strain: 98900666 DSS DV-2	TYEPDVDLGSGTRNIGIESEIPNLDIIGKRIEKIKQEHETSWHYDQDHPYKTWAYHGSYETKQTGSASSMVNGVVRLLTK	[80]
FJ654700.1 Yellow fever virus 17D/Tiantan complete genome	TLEADVILPIGTRSVETDKGPLDKEAIEERVERIKSEYMTSWFYDNDNPYRTWHYCGSYVTKTSGSAASMVNGVIKILTY	[80]
GQ379163.1 Yellow fever virus strain case 2 complete genome	TLEADVILPIGTRSVETDKGPLDKEAIEERVERIKSEYMTSWFYDNDNPYRTWHYCGSYVTKTSGSAASMVNGVIKILTY	[80]
Zika virus strain ZikaSPH2015 complete genome	PWDVVTGVTGIAM DTTPYGQQRVFKEKVDTRVPDPQEGTRQVMSMVSSWLWKELGKHKRPRVCTKEEF---INKVRSNA	[160]
Dengue virus 2 isolate DENV-2/PK complete genome	PWDVIPMVTQMAMTDTTPFGQQRVFKEKVDTRTQEPKEGTKKLMKITAEWLWKELGKKKTPRMCTREEF---ARKVRSNA	[160]
Hepatitis C virus subtype 2a isolate PR63cc complete genome	------------------FDRMQVLDTYYDSVLKDVKLAASKVS---ARLLTLEEACQLTPPHSARSKYGFGAKEVRSLS	[160]
Hepatitis C virus subtype 1b isolate HCV-1b/US/BID-V148/2004 complete genome	------------------FDRLQVLDDHYRDVLKEMKAKASTVK---AKLLSVEEACRLTPPHSARSKFGYGAKDVRNLS	[160]
Hepatitis C virus subtype 3a isolate ILBSRAS UG polyprotein gene complete cds	------------------FDRLQVLDDHYKTTLKEVKERASRVK---ARMLTIEEACALVPPHSARSKFGYSAKDVRSLS	[160]
Hepatitis C virus subtype 4a isolate 25 polyprotein gene complete cds	------------------FDRLQVVDNHYNETLKEIKARASRVK---ARLLTTEEACDLTPPHSARSKFGYGAKDVRSHS	[160]
AB189124.1 Dengue virus 2 genomic RNA complete genome strain: 98900666 DSS DV-2	PWDVIPMVTQMAMTDTTPFGQQRVFKEKVDTRTQEPKEGTKKLMKITAEWLWKELGKKKTPRMCTREEF---TRKVRSNA	[160]
FJ654700.1 Yellow fever virus 17D/Tiantan complete genome	PWDRIEEVTRMAMTDTTPFGQQRVFKEKVDTRAKDPPAGTRKIMKVVNRWLFRHLAREKNPRLCTKEEF---IAKVRSHA	[160]
GQ379163.1 Yellow fever virus strain case 2 complete genome	PWDRIEEVTRMAMTDTTPFGQQRVFKEKVDTRAKDPPAGTRKIMKVVNRWLFRHLAREKSPRLCTKEEF---IAKVRSHA	[160]
Zika virus strain ZikaSPH2015 complete genome	ALGAIFEEEKEWKTAVEAVNDPRFWALVDKEREHHLRGECQSCVYNMMGKREKKQGEFGKAKGSRAIWYMWLGAR-FLEF	[240]
Dengue virus 2 isolate DENV-2/PK complete genome	ALGAIFTDENKWKSAREAVEDSRFWELVDKERNLHLEGKCETCVYNMMGKREKKIGEFGKAKGSRAVWYMWLGAR-FLEF	[240]
Hepatitis C virus subtype 2a isolate PR63cc complete genome	G-RAVHHIKSVWEDLLEDSQTPIPTTIMAKTEVF--------CVDPTKGGK----------KAARLIVYPDLSVR-VCEK	[240]
Hepatitis C virus subtype 1b isolate HCV-1b/US/BID-V148/2004 complete genome	S-KAVNHIHSVWKDLLEDTETPIDTTIMAKNEVF--------CVQPEKGGR----------KPARLIVFPDLGVR-VCEK	[240]
Hepatitis C virus subtype 3a isolate ILBSRAS UG polyprotein gene complete cds	S-KAINQIRSVWEDLLEDTTTPIPTTIMAKNEVF--------CVDPVKGGR----------KPARLIVYPDLGVR-VCEK	[240]
Hepatitis C virus subtype 4a isolate 25 polyprotein gene complete cds	R-KAVNHINSVWEDLLEDNNTPIPTTIMAKNEVF--------AVNPAKGGR----------KPARLIVYPDLGGPGFARR	[240]
AB189124.1 Dengue virus 2 genomic RNA complete genome strain: 98900666 DSS DV-2	ALGAIFTDENKWKSAREAVEDSGFWELVDKERNLHLEGKCETCVYNMMGKREKKLGEFGKAKGSRAIWYMWLGAR-FLEF	[240]
FJ654700.1 Yellow fever virus 17D/Tiantan complete genome	AIGAYLEEQEQWKTANEAVQDPKFWELVDEERKLHQQGRCRTCVYNMMGKREKKLSEFGKAKGSRAIWYMWLGAR-YLEF	[240]
GQ379163.1 Yellow fever virus strain case 2 complete genome	AIGAYLEEQEQWKTANEAVQDPKFWELVDEERKLHQQGRCRTCVYNMMGKREKKLSEFGKAKGSRAIWYMWLGAR-YLEF	[240]
Zika virus strain ZikaSPH2015 complete genome	EALGFLNEDHWMGRENSGGGVEGLGLQRLGYVLEEMSRIPGGRMYADDTAGWDTRISRFDLENEALITN--QMEKGHRAL	[320]
Dengue virus 2 isolate DENV-2/PK complete genome	EALGFLNEDHWFSRENSLSGVEGEGLHKLGYILREVSKKEGGAMYADDTAGWDTRITLEDLKNEEMVTN--HMEGEHKKL	[320]
Hepatitis C virus subtype 2a isolate PR63cc complete genome	MALYDVTQKLPQAVMGASYGFQYSPAQRVEFLLKAWADKKDPMGFSYDTRCFDSTVTERDIRTEESIYQACSLPEEARTA	[320]
Hepatitis C virus subtype 1b isolate HCV-1b/US/BID-V148/2004 complete genome	MALYDVVSTLPQAVMGSSYGFQYSPGQRVEFLVNAWKSKKNPMGFSYDTRCFDSTVTESDIRVEESIYQCCDLAPEARQA	[320]
Hepatitis C virus subtype 3a isolate ILBSRAS UG polyprotein gene complete cds	RALYDVIQKLSIETMGPAYGFQYSPQQRVERLLKMWTSKKTPLGFSYDTRCFDSTVTEQDIRVEEEIYQCCDLEPEARKV	[320]
Hepatitis C virus subtype 4a isolate 25 polyprotein gene complete cds	EHFTTSSAQLPKAVMGAAYGFQYSPAQRVEFLLTSWKSKNTPMGFSYDTRCFDSTVTEKDIRTEEEVYQCCDLEPEARKV	[320]
AB189124.1 Dengue virus 2 genomic RNA complete genome strain: 98900666 DSS DV-2	EALGFLNEDHWFSRENSLSGVEGEGLHKLGYILRDVSKKEGGAMYADDTAGWDTRITLEDLKNEEMVTN--HMEGEHKKL	[320]
FJ654700.1 Yellow fever virus 17D/Tiantan complete genome	EALGFLNEDHWASRENSGGGVEGIGLQYLGYVIRDLAAMDGGGFYADDTAGWDTRITEADLDDEQEILN--YMSPHHKKL	[320]
GQ379163.1 Yellow fever virus strain case 2 complete genome	EALGFLNEDHWASRENSGGGVEGIGLQYLGYVIRDLAAMDGGGFYADDTAGWDTRITEADLDDEQEILN--YMSPHHKKL	[320]
Zika virus strain ZikaSPH2015 complete genome	ALAIIKYTYQNKVVKVLRPAEKGKTVMDIISRQDQRGSGQVVTYALNTFTNLVVQLIRNMEAEEVLEMQDLWLLRRS--E	[400]
Dengue virus 2 isolate DENV-2/PK complete genome	AEAIFKLTYQNKVVRVQRPTPRG-TVMDIISRRDQRSSGQVGTYGLNTFTNMGAQLIRQMEGEGVFKGIQHLTATEE--V	[400]
Hepatitis C virus subtype 2a isolate PR63cc complete genome	IHSLTERLYVGGPMLNSKGQTCG--------YRRCRASGVLTTSMGNTITCY-------VKALAACKAAGIVAPT-----	[400]
Hepatitis C virus subtype 1b isolate HCV-1b/US/BID-V148/2004 complete genome	IRSLTERLYIGGPLTNSKGQNCG--------YRRCRASGVLTTSCGNTLTCY-------LKASAACRAAKLQDCT-----	[400]
Hepatitis C virus subtype 3a isolate ILBSRAS UG polyprotein gene complete cds	ISSLTERLYCGGPMFNSKGAPCG--------YRRCRASGVLPTSFGNTITCY-------IKASAAAKAAGLRNPD-----	[400]
Hepatitis C virus subtype 4a isolate 25 polyprotein gene complete cds	IAALTERLYVGGPMHNSKGDLCG--------YRRCRASGVYTTSFGNTLTCY-------LKATAAIKAGRAEDCT-----	[400]
AB189124.1 Dengue virus 2 genomic RNA complete genome strain: 98900666 DSS DV-2	AEAIFKLTYQNKVVRVQRPTPRG-TVMDIISRRDQRGSGQVVTYGLNTFTNMEAQLIRQMEGEGVFKSIQHLTVTEE--I	[400]
FJ654700.1 Yellow fever virus 17D/Tiantan complete genome	AQAVMEMTYKNKVVKVLRPAPGGKAYMDVISRRDQRGSGQVVTYALNTITNLKVQLIRMAEAEMVIHHQHVQDCDESVLT	[400]
GQ379163.1 Yellow fever virus strain case 2 complete genome	AQAVMEMTYKNKVVKVLRPAPGGKAYMDVISRRDQRGSGQVVTYALNTITNLKVQLIRMAEAEMVIHHQHVQDCDESVLH	[400]
Zika virus strain ZikaSPH2015 complete genome	KVTNWLQSNGWDRLKRMAVSGDDCVV----KPIDDRFAHALRFLNDMGKVRKDTQEWKPSTGWDNWEEVPFCSHHFNKLH	[480]
Dengue virus 2 isolate DENV-2/PK complete genome	AVQDWLARVGRERLSRMAISGDDCVV----KPLDDRFASALTALNDMGKVRKDIQQWEPSRGWNDWTQVPFCSHHFHELI	[480]
Hepatitis C virus subtype 2a isolate PR63cc complete genome	----------------MLVCGDDLVVISESQGTEEDERNLRAFTEAMTRYSAPPGD--PPRPEYDLELITSCSSNVSVAL	[480]
Hepatitis C virus subtype 1b isolate HCV-1b/US/BID-V148/2004 complete genome	----------------MLVCGDDLVVICESAGTHEDAASLRVFTEAMTRYSAPPGD--PPQPEYDLELITSCSSNVSVAH	[480]
Hepatitis C virus subtype 3a isolate ILBSRAS UG polyprotein gene complete cds	----------------FLVCGDDLVVGAESEGVEEDRAALRAFTEAMTRYSAPPGD--APQPTYDLELITSCSSNVSVAR	[480]
Hepatitis C virus subtype 4a isolate 25 polyprotein gene complete cds	----------------MLVCGDDLVVIAESGGVDEDNRALRAFTEAMTRYSAPPGD--APQPAYDLELITSCSSNVSVAH	[480]
AB189124.1 Dengue virus 2 genomic RNA complete genome strain: 98900666 DSS DV-2	AVKNWLARVGRERLSRMAISGDDCVV----KPLDDRFASALTALNDMGKVRKDIQQWEPSRGWNDWTQVPFCSHHFHELI	[480]
FJ654700.1 Yellow fever virus 17D/Tiantan complete genome	RLEAWLTEHGCNRLKRMAVSGDDCVV----RPIDDRFGLALSHLNAMSKVRKDISEWQPSKGWNDWENVPFCSHHFHELQ	[480]
GQ379163.1 Yellow fever virus strain case 2 complete genome	RLEAWLTEHGCNRLKRMAVSGDDCVV----RPIDDRFGLALSHLNAMSKVRKDISEWQPSKGWNDWENVPFCSHHFHELQ	[480]
Zika virus strain ZikaSPH2015 complete genome	LKDGRSIVVPCRHQDELIGRA-----RVSPGAGWSIRETACLAKSYAQMWQLLYFH---------RRDLRLMANAICSSV	[560]
Dengue virus 2 isolate DENV-2/PK complete genome	MKDGRVLVVPCRNQDELIGRA-----RISQGAGWSLRETACLGKSYAQMWSLMYFH---------RRGLRLAANAICSAV	[560]
Hepatitis C virus subtype 2a isolate PR63cc complete genome	GPRGGRRYYLTRDPTTPLARAAWETVRHSPVNSWLGNIIQYAPTIWVRMVLMTHFFSILMAQDTLDQNLNFEMYGAVYSV	[560]
Hepatitis C virus subtype 1b isolate HCV-1b/US/BID-V148/2004 complete genome	DATGKRVYYLTRDPTTPLARAAWETARHTPVNSWLGNIIMYAPTLWARMILMTHFFSILLAQEQLEKALDCQIYGACYSI	[560]
Hepatitis C virus subtype 3a isolate ILBSRAS UG polyprotein gene complete cds	DDKGKRYYYLTRDATTPLARAAWETARHTPVNSWLGNIIMYAPTIWVRMVMMTHFFSILQSQEILDRPLDFEMYGATYSV	[560]
Hepatitis C virus subtype 4a isolate 25 polyprotein gene complete cds	DATGKKVYYLTRDPETPLARAAWETVRHTPVNSWLGNIIVYAPTIWVRMVLMTHFFSILQSQEALEKALDFDMYGVTYSI	[560]
AB189124.1 Dengue virus 2 genomic RNA complete genome strain: 98900666 DSS DV-2	MKDGRVLVVPCRNQDELIGRA-----RISQGAGWSLRETACLGKSYAQMWSLMYFH---------RRDLRLAANAICSAV	[560]
FJ654700.1 Yellow fever virus 17D/Tiantan complete genome	LKDGRRIVVPCREQDELIGRG-----RVSPGNGWMIKETACLSKAYANMWSLMYFH---------KRDMRLLSLAVSSAV	[560]
GQ379163.1 Yellow fever virus strain case 2 complete genome	LKDGRRIVVPCREQDELIGRG-----RVSPGNGWMIKETACLSKAYANMWSLMYFH---------KRDMRLLSLAV?SAV	[560]
Zika virus strain ZikaSPH2015 complete genome	PVDWVPT------GRTTWSIHG--KGEWMTTEDMLVVWN----RVWIEENDHMEDKTPVTKWTDIPYLGKREDLWCGSLI	[640]
Dengue virus 2 isolate DENV-2/PK complete genome	PSHWVPT------SRTTWSIHA--RHEWMTTEDMLTVWN----RVWIQENPWMEDKTPVESWEEIPYLGKRGDQWCGSLI	[640]
Hepatitis C virus subtype 2a isolate PR63cc complete genome	SPLDLPAIIERLHGLDAFSLHTYSPHELTRVAAALRKLGAPPLRVWKSRARAVRAS--------LIFRGGRAAV-CGRYL	[640]
Hepatitis C virus subtype 1b isolate HCV-1b/US/BID-V148/2004 complete genome	EPLDLPQIIQRLHGLSAFSLHSYSPGEINRVASCLRKLGVPPLRVWRHRARGVRAK--------LLSQGGRAAT-CGKYL	[640]
Hepatitis C virus subtype 3a isolate ILBSRAS UG polyprotein gene complete cds	TPLDLPAIIERLHGLSAFTLHSYSPVELNRVAGTLRKLGCPPLRAWRHRARAVRAK--------LIAQGGKAKI-CGLYL	[640]
Hepatitis C virus subtype 4a isolate 25 polyprotein gene complete cds	TPLDLPAIIQRLHGLSAFTLHGYSPHELNRVAGSLRKLGVPPLRAWRHRARAVRAK--------LIAQGGRAKI-CGIYL	[640]
AB189124.1 Dengue virus 2 genomic RNA complete genome strain: 98900666 DSS DV-2	PSHWVPT------SRTTWSIHA--THEWMTTEDMLTVWN----RVWIQENPWMEDKTPVESWEEIPYLGKREDQWCGSLI	[640]
FJ654700.1 Yellow fever virus 17D/Tiantan complete genome	PTSWVPQ------GRTTWSIHG--KGEWMTTEDMLEVWN----RVWITNNPHMQDKTMVKKWRDVPYLTKRQDKLCGSLI	[640]

GQ379163.1 Yellow fever virus strain case 2 complete genome	P T S W V P Q - - - - - - G R T T W S I H G - - K G E W M T T E D M L E V W N - - - - R V W I T N N P H M Q D K T M V K K W R D V P Y L T K ? Q D K L C G S L I	[640]
Zika virus strain ZikaSPH2015 complete genome	G H R P R T T W A E N I K N T V N M V R R I - - - - - - - - - - - - I G D E E K Y M D Y L S T Q V R Y L G E E G S T P G V L * A P I L M L S G L L V S H S L G K	[720]
Dengue virus 2 isolate DENV-2/PK complete genome	G L T S R A T W A R N I Q T A I N R V R S L - - - - - - - - - - - - I G - S E G Y T D Y M P S M K R F R R E E D E A G V L W * K A E L T * D K I R S Q V G L S H	[720]
Hepatitis C virus subtype 2a isolate PR63cc complete genome	- - - - - F N W A V K T K L K L T P L P E A R L L D L S S W F T V G A G G G D I Y H S V S R A R P R L L - - - - - - - - - - - - - - - L L S L L L L S V G V G L	[720]
Hepatitis C virus subtype 1b isolate HCV-1b/US/BID-V148/2004 complete genome	- - - - - F N W A V R T K L K L T P I P A A S R L D L S N W F V A G Y S G G D I Y H S V S H A R P R W F - - - - - - - - - - - - - - - M L C L L L L S V G V G I	[720]
Hepatitis C virus subtype 3a isolate ILBSRAS UG polyprotein gene complete cds	- - - - - F N W A V R T K A K L T P L P A A G Q L D L S S W F T V G V G G N D I Y H S V S R A R T R H L - - - - - - - - - - - - - - - L L C L L L L T V G V G I	[720]
Hepatitis C virus subtype 4a isolate 25 polyprotein gene complete cds	- - - - - F N W A V K T K L K L T P L P A A A N L D L S S W F T V G A G G G D I Y H S V S R A R P R Y L - - - - - - - - - - - - - - - L L C L L L L S V G V G I	[720]
AB189124.1 Dengue virus 2 genomic RNA complete genome strain: 98900666 DSS DV-2	G L T S R A T W A K N I Q T A I N Q V R S L - - - - - - - - - - - - I G -	[720]
FJ654700.1 Yellow fever virus 17D/Tiantan complete genome	G M T N R A T W A S H I H L V I H R I R T L - - - - - - - - - - - - I G -	[720]
GQ379163.1 Yellow fever virus strain case 2 complete genome	G M T N R A T W A S H I H L V I H R I R T L - - - - - - - - - - - - I G -	[720]
Zika virus strain ZikaSPH2015 complete genome	A V Q P V T P P G E A G K P S L * S G R E R H G T E E A M L P V S P S E D T E S K N P T R L E A Q D G - - - - - - - - - - K R R W R P S P P F N L G P E L E I S	[800]
Dengue virus 2 isolate DENV-2/PK complete genome	S T E K T M L P V S P V Q G R * K K S G H Y K C H S L S K L C S L * L H L G R C K K S G R P Q T M E A V R M A * W T S G * R R P L P C K S Q Q Q W G P K V R * S	[800]
Hepatitis C virus subtype 2a isolate PR63cc complete genome	F L L P A R * S G A H W L H S I A N C F F F F F F F F F F F F F P F L F F L F P S Y P T L L S F L V A - - - - - - - - - - - - - - - - - - - P S * P * S R L A	[800]
Hepatitis C virus subtype 1b isolate HCV-1b/US/BID-V148/2004 complete genome	Y L L P N R * T G ? -	[800]
Hepatitis C virus subtype 3a isolate ILBSRAS UG polyprotein gene complete cds	F L L P A R * A G R I T -	[800]
Hepatitis C virus subtype 4a isolate 25 polyprotein gene complete cds	F L L P A R * -	[800]
AB189124.1 Dengue virus 2 genomic RNA complete genome strain: 98900666 DSS DV-2	- -	[800]
FJ654700.1 Yellow fever virus 17D/Tiantan complete genome	- -	[800]
GQ379163.1 Yellow fever virus strain case 2 complete genome	- -	[800]
Zika virus strain ZikaSPH2015 complete genome	C G S P E E G L V V R G -	[856]
Dengue virus 2 isolate DENV-2/PK complete genome	C N L T G R T R G * R R P P R N K K Q H I D A G K D Q R S C C L ? -	[856]
Hepatitis C virus subtype 2a isolate PR63cc complete genome	V K G P * A A * L Q R V P * L V S L Q I M ? -	[856]
Hepatitis C virus subtype 1b isolate HCV-1b/US/BID-V148/2004 complete genome	- -	[856]
Hepatitis C virus subtype 3a isolate ILBSRAS UG polyprotein gene complete cds	- -	[856]
Hepatitis C virus subtype 4a isolate 25 polyprotein gene complete cds	- -	[856]
AB189124.1 Dengue virus 2 genomic RNA complete genome strain: 98900666 DSS DV-2	- -	[856]
FJ654700.1 Yellow fever virus 17D/Tiantan complete genome	- -	[856]
GQ379163.1 Yellow fever virus strain case 2 complete genome	- -	[856]

Footnote: Conserved regions highlighted in yellow and motifs A to E with red letters
